# An end‐to‐end AI‐based framework for automated discovery of rapid CEST/MT MRI acquisition protocols and molecular parameter quantification (AutoCEST)

**DOI:** 10.1002/mrm.29173

**Published:** 2022-01-28

**Authors:** Or Perlman, Bo Zhu, Moritz Zaiss, Matthew S. Rosen, Christian T. Farrar

**Affiliations:** ^1^ Athinoula A. Martinos Center for Biomedical Imaging, Department of Radiology Massachusetts General Hospital and Harvard Medical School Charlestown MA USA; ^2^ Magnetic Resonance Center Max Planck Institute For Biological Cybernetics Tübingen Germany; ^3^ Department of Neuroradiology University Hospital Erlangen Friedrich‐Alexander‐Universität Erlangen‐Nürnberg (FAU) Erlangen Germany; ^4^ Department of Physics Harvard University Cambridge MA USA

**Keywords:** chemical exchange saturation transfer (CEST), deep learning, magnetic resonance fingerprinting (MRF), magnetization transfer (MT), optimization, quantitative imaging

## Abstract

**Purpose:**

To develop an automated machine‐learning‐based method for the discovery of rapid and quantitative chemical exchange saturation transfer (CEST) MR fingerprinting acquisition and reconstruction protocols.

**Methods:**

An MR physics‐governed AI system was trained to generate optimized acquisition schedules and the corresponding quantitative reconstruction neural network. The system (termed AutoCEST) is composed of a CEST saturation block, a spin dynamics module, and a deep reconstruction network, all differentiable and jointly connected. The method was validated using a variety of chemical exchange phantoms and in vivo mouse brains at 9.4T.

**Results:**

The acquisition times for AutoCEST optimized schedules ranged from 35 to 71 s, with a quantitative image reconstruction time of only 29 ms. The resulting exchangeable proton concentration maps for the phantoms were in good agreement with the known solute concentrations for AutoCEST sequences (mean absolute error = 2.42 mM; Pearson’s r=0.992, p<0.0001), but not for an unoptimized sequence (mean absolute error = 65.19 mM; Pearson’s r=‐0.161, p=0.522). Similarly, improved exchange rate agreement was observed between AutoCEST and quantification of exchange using saturation power (QUESP) methods (mean absolute error: 35.8 Hz, Pearson’s r=0.971, p<0.0001) compared to an unoptimized schedule and QUESP (mean absolute error = 58.2 Hz; Pearson’s r=0.959, p<0.0001). The AutoCEST in vivo mouse brain semi‐solid proton volume fractions were lower in the cortex (12.77% ± 0.75%) compared to the white matter (19.80% ± 0.50%), as expected.

**Conclusion:**

AutoCEST can automatically generate optimized CEST/MT acquisition protocols that can be rapidly reconstructed into quantitative exchange parameter maps.

## 1 INTRODUCTION

Chemical exchange saturation transfer (CEST) is an increasingly explored molecular imaging technique which allows for the detection of signals associated with milli‐molar concentrations of proteins, metabolites, and various molecular compounds.^1^, ^2^ It uses frequency selective radiofrequency (RF) pulses to saturate the magnetization of exchangeable protons on proteins, lipids, and other biologically interesting compounds that later undergo chemical exchange with the bulk water protons, thus altering the MR‐detectable signal.^3^

The potential benefit of using the CEST contrast mechanism was demonstrated in a variety of clinical applications, including cancer detection and grading,^4^ stroke characterization,^5^ characterization of neurodegenerative disorders,^6^ kidney disease monitoring,^7^, ^8^ cartilage and intervertebral disc imaging,^9^, ^10^ cell tracking,^11^, ^12^, ^13^ and cardiac disease assessment.^14^

The most common analysis method for CEST‐weighted imaging is the magnetization transfer ratio asymmetry (${\text{MTR}}_{\mathrm{asym}}$). Although it is straightforward to calculate and was found useful in many reports, this metric is affected by a mixed contribution from several exchange and relaxation properties, such as the relayed aliphatic nuclear Overhauser enhancement (rNOE) and the water $T_{1}$ relaxation time, that may bias the interpretation of the obtained contrast.^15^ Moreover, the ${\text{MTR}}_{\mathrm{asym}}$ is strongly affected by the saturation pulse parameters used, challenging the comparison of findings obtained using different protocols, and requiring a rigorous optimization of the acquisition parameters.^16^

A quantitative CEST technique would clearly be beneficial for overcoming the abovementioned challenges. The exchange parameters (proton volume fraction and chemical exchange rate) can be quantified by acquiring multiple Z‐spectra with different saturation pulse durations and/or powers, followed by analysis using methods such as quantification of exchange using saturation power/time (QUESP/QUEST),^17^ Omega‐plot,^18^, ^19^ or a full fitting of the Bloch–McConnell equations.^20^ However, the long acquisition times and the complexity of the in vivo multipool environment render this approach suboptimal for routine clinical use. CEST MR‐fingerprinting (MRF^21^) is a recently suggested promising alternative.^22^, ^23^, ^24^ In the MR fingerprinting approach, a pseudo random and fast CEST acquisition schedule is used to obtain different “signal‐signatures,” representing different combinations of solute concentration and chemical exchange rate. The acquired experimental signals are then compared to a simulated signal dictionary, allowing the generation of quantitative CEST parameter maps. However, the CEST‐MRF performance, and ability to discriminate different exchange rates and proton volume fractions, is critically dependent on the acquisition parameter schedule used.^25^ This mandates a careful optimization of the imaging protocol, which is very challenging for CEST/MT imaging given the large number of exchangeable proton pools involved.

The purpose of this work is to develop and validate a novel paradigm for conducting and analyzing CEST experiments. We hypothesized that an MR physics governed AI system, termed here as AutoCEST, can be designed and trained to simultaneously generate an optimized and fast CEST acquisition schedule and at the same time provide the means for reconstructing quantitative exchange‐parameter maps, for any given and broadly defined multi‐pool CEST/MT scenario. To demonstrate the efficiency and robustness of the method, a validation study using a variety of different CEST phantoms was performed, followed by an in vivo mouse imaging experiment.

## 2 METHODS

### 2.1 AutoCEST architecture and realization

**FIGURE 1:**
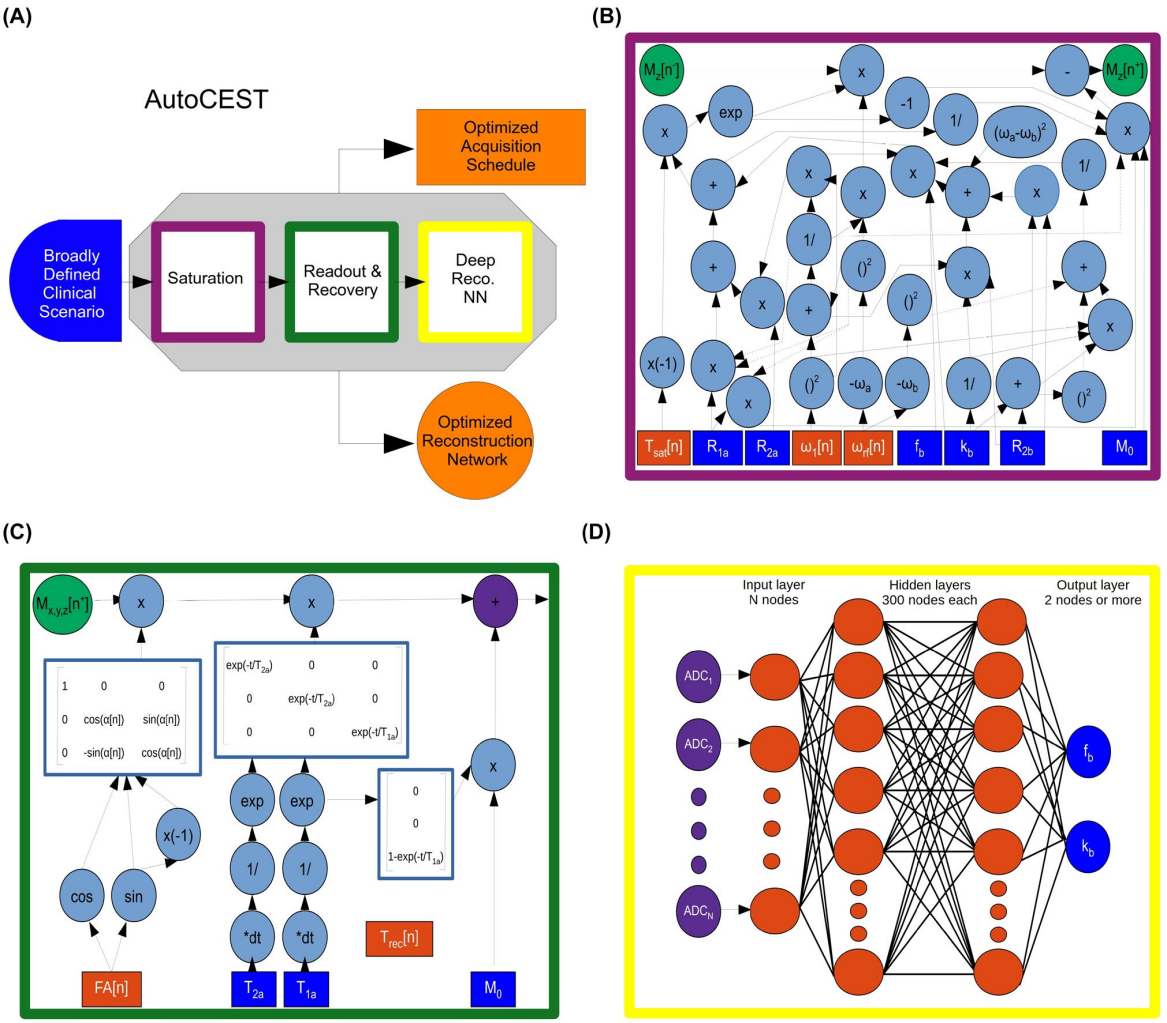
A, Schematic representation of the AutoCEST pre‐experiment pipeline. A broadly defined clinical scenario serves as input which allows the experiment optimization by sequentially simulating CEST saturation (purple), readout and recovery (green), and deep reconstruction (yellow). AutoCEST outputs an optimized acquisition schedule and a reconstruction network (orange). B, CEST saturation block as a computational graph. The blue rectangles represent the input tissue parameters: initial magnetization ($M_{0}$), water relaxation rates ($R_{1a}$, $R_{2a}$), solute transverse relaxation ($R_{2b}$), exchange‐rate ($k_{b}$), and volume fraction ($f_{b}$). The orange rectangles represent the dynamically updated protocol parameters: saturation time ($T_{\mathrm{sat}}$), saturation power ($\omega_{1}$), saturation frequency offset ($\omega_{\mathrm{rf}}$). The graph calculates the magnetization at the end of the saturation block $M_{z}\left[n^{+}\right]$. C, Bloch equation‐based image readout as a computational graph. The blue rectangles represent the water‐pool parameters, while the orange rectangles represent the dynamically updated protocol parameters: flip angle (FA) and recovery time ($T_{\mathrm{rec}}$), which is embedded in the appropriate relaxation step. Note that this is a partial display due to space limitations. D, Deep reconstruction network for decoding the “ADC” MR signals (purple circles), obtained in C into CEST quantitative parameters ($f_{b}$ and $k_{b}$, blue circles)

An overview of the AutoCEST approach is described in Figure 1A. For each chemical exchange scenario of interest (e.g., amide, amine, creatine, magnetization transfer (MT), etc.), the system gets as input a general description of the expected range of parameter values and simulates the expected MR signals from a random CEST acquisition protocol. The system then performs automatic optimization, which ultimately outputs a refined set of acquisition protocol parameters (Figure 1B,C orange rectangles) as well as optimized neural network weights (Figure 1D, orange circles), capable of transforming the measured signals into quantitative CEST/MT proton exchange parameter maps.

The proposed technique is based on the integration of CEST physics and spin dynamics with deep learning. In a classic neural network, each of the nodes contains a “weight element,” which is updated and optimized during the backward propagation step. To allow an analogous equivalent update of the CEST experiment parameters and achieve efficient optimization using auto‐differentiation, the analytical solution of the governing spin dynamics for every step of the imaging experiment was represented as a computational graph (Figure 1B,C). Next, a deep reconstruction network^26^ was used to obtain quantitative CEST/MT parameter maps (proton volume fraction and exchange rate). Notably, the acquisition and reconstruction steps are serially connected to allow joint optimization using automatic differentiation and stochastic gradient descent. The detailed AutoCEST steps include:

#### 2.1.1 CEST saturation block

The analytical solution of the Bloch–McConnell equations for continous wave RF irradiation, for either a two‐pool^27^ (water and solute proton pools) or a three‐pool^20^ (water, solute, and semi‐solid/MT proton pools) imaging scenario was represented as a computational graph (Figure 1B). This allows the calculation of the water‐pool $M_{z}$ component at the end of the saturation, and more importantly, the update of the saturation‐block parameters (Figure 1B, orange rectangles) during training.

#### 2.1.2 Readout and relaxation spin dynamics module

In the next step of the forward‐direction modeling, the transverse spin components are zeroed‐out, assuming sufficient gradient spoiling is applied. Next, the spin dynamics are calculated during excitation and relaxation, using the Bloch equations with a discrete‐time state‐space model in the rotating frame ^28^ (Figure 1C). This allows for the update of the flip‐angle (FA) and the recovery time ($T_{\mathrm{rec}}$) parameters as well as the calculation of the expected “ADC” signals.

#### 2.1.3 Deep reconstruction network

The resulting MR signals are two‐norm normalized along the temporal dimension in a pixel‐wise manner and mapped into CEST quantitative parameters using a fully connected four‐layer deep reconstruction network^26^ (Figure 1D). The neural network is composed of a series of fully connected dense layers, with two hidden layers of 300 nodes each and activated by hyperbolic tangent (tanh) functions.

The entire pipeline was implemented using PyTorch 1.0.1 and Python 3.6.8 on a Linux laptop computer equipped with an 8‐core Intel i7‐7700HQ CPU (2.80 GHz). AutoCEST was trained for a variety of chemical exchange scenarios as described in Sections 2.2, 2.4.2, and Supporting Information Table S1. For each scenario, acquisition schedules of N=10 raw (molecular information encoding) images were generated. The batch size was set to 256 and the number of training epochs set to 100,^29^ while a different development set of simulated signals (not included in the training data) was used to confirm that over‐fitting is not reached. To further promote robust learning, white Gaussian noise (standard deviation of 0.002) was injected into the training data.^30^, ^31^ The loss was defined as the mean‐squared‐error between the estimated proton exchange rate and volume fraction values and their corresponding ground‐truth values. The RMSprop algorithm^32^ was used as the optimizer, with the learning rates of the acquisition schedule parameters and the reconstruction network set to 0.001 and 0.0001, respectively.

To provide basic intuition on the optimization process, AutoCEST was set to update only the saturation pulse power for some of the scenarios (iohexol, BSA, and in vivo amide). Next, 2,3, or 5 different acquisition parameters were defined in a simultaneous parameter optimization for the in vivo MT, pCr, and L‐arginine scenarios, respectively.

**FIGURE 2:**
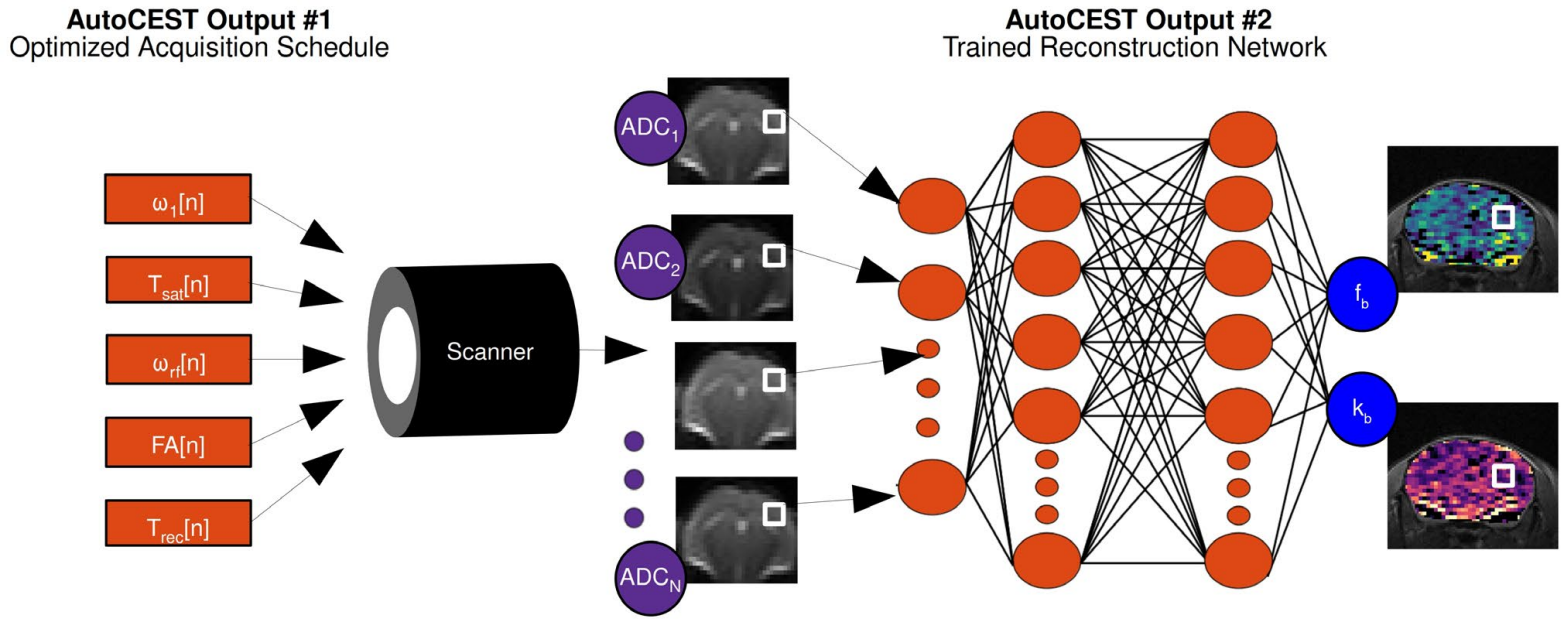
AutoCEST‐based quantitative image reconstruction. The optimized protocol parameters (orange rectangles, $\omega_{1}$ = saturation pulse power, $T_{\mathrm{sat}}$ = saturation pulse duration, $\omega_{\mathrm{rf}}$ = saturation pulse frequency offset, FA = readout flip angle, $T_{\mathrm{rec}}$= recovery time) are loaded into the scanner, allowing for the acquisition of N raw ADC (molecular information encoding) images. The images are fed voxelwise into the trained reconstruction network (orange circles), resulting in quantitative CEST/MT parameter maps (e.g., proton volume fraction $f_{b}$ and exchange rate $k_{b}$)

Finally, the optimal acquisition schedule parameters found by AutoCEST are loaded into the MR scanner, and a set of N, molecular information encoding, raw images are acquired (Figure 2). The resulting images are then fed voxel‐wise into the AutoCEST‐trained reconstruction network, resulting in quantitative CEST/MT maps of the imaged subject.

### 2.2 Phantom preparation

To validate the suggested approach, an extensive in vitro imaging study was performed using a set of seven imaging phantoms, each composed of three different vials of a particular CEST compound, dissolved in PBS or in a buffer titrated to a particular pH value between 4.0 and 7.4. The compound concentrations were varied between 12.5 and 100 mM in all cases except for BSA, where the w/w concentration was varied between 7.5% and 15%.^33^, ^34^, ^35^, ^36^ To verify the AutoCEST robustness for various imaging scenarios, the following compounds were used:

#### 2.2.1 Iohexol

An x‐ray iodinated contrast agent, used as a CEST agent for extracellular pH quantification. Iohexol contains two exchangeable amide protons at a chemical shift of 4.3 ppm relative to the resonance frequency of water.^37^, ^38^

#### 2.2.2 Phosphocreatine (pCr)

A crucial metabolite for heart and skeletal muscle energetics, contains a single guanidinium exchangeable proton at 2.6 ppm.^31^, ^39^, ^40^

#### 2.2.3 L‐arginine

An amino acid with three equivalent exchangeable amine protons with a chemical shift of 3 ppm with respect to the water resonance.

#### 2.2.4 Bovine serum albumin (BSA)

A protein with a large number of exchangeable amide (3.5 ppm), amine (∼2.75 ppm), and rNOE (∼‐3.5 ppm) protons.

While iohexol, pCr, and L‐arginine contain additional exchangeable protons at other chemical shifts than mentioned above, the optimization was focused on their commonly targeted exchangeable protons. To demonstrate the ability of detecting multiple CEST targets within the same phantom, various AutoCEST‐based acquisition schedules were generated for imaging the amide, amine, and rNOE exchangeable protons of BSA.

### 2.3 Animal preparation

All animal experiments and procedures were performed in accordance with the NIH Guide for the Care and Use of Laboratory Animals and were approved by the Institutional Animal Care and Use Committee of the Massachusetts General Hospital. Three C57/BL6 wild‐type male mice (27–31 gr) were purchased from Jackson Laboratory. They were anesthetized using 1%–2% isoflurane and placed on an MRI cradle with ear and bite bars to secure the head. Respiration rate was monitored with a small animal physiological monitoring system (SA Instruments, Stony Brook, NY), and the temperature was maintained by blowing warm air in the bore of the magnet.

### 2.4 Magnetic resonance imaging

All imaging experiments were conducted using a 9.4T MRI scanner (Bruker Biospin, Billerica, MA), employing an in‐house programmed, flexible CEST‐EPI protocol,^22^, ^25^, ^41^ loaded with the acquisition parameters generated by AutoCEST.

#### 2.4.1 Phantom studies

**FIGURE 3:**
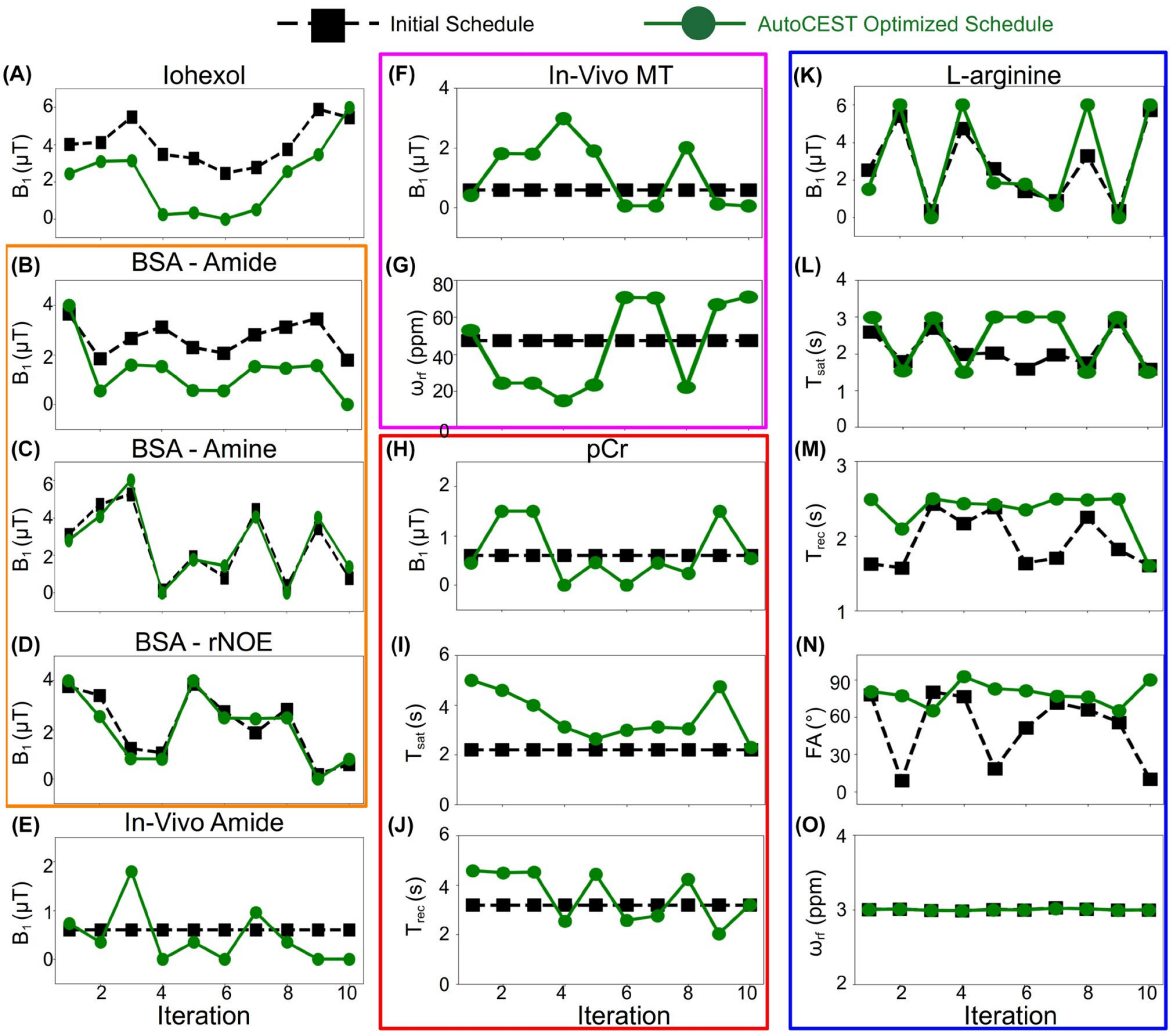
AutoCEST‐generated acquisition schedules for the various imaging scenarios studied. The black dashed lines with squares represent the random/fixed parameters used for initializing the optimization and the green lines with circles represent the final AutoCEST‐optimized schedule. For iohexol (A), BSA, amide (B), BSA, amine (C), BSA, rNOE (D), and in vivo amide (E), only the saturation pulse power ($B_{1}$) was optimized. For the In vivo MT (F,G), pCr (H–J), and L‐arginine (K–O) cases, 2, 3, and 5 acquisition parameters were simultaneously optimized, respectively. Additional acquisition schedule information is available in Supporting Information Table S1

Imaging was performed using a transmit/receive volume coil (Bruker Biospin, Billerica, MA), a field of view (FOV) of 32 × 32 mm^2^, a matrix of 64 × 64 pixels, and a 5 mm slice thickness. The iohexol and L‐arginine phantoms were imaged at room temperature. The pCr and BSA phantoms were heated to $37^{\circ }$C, using a feedback loop between a small animal physiological monitoring system (SA Instruments, Stony Brook, NY) and a warm air blower. Each phantom was imaged using the AutoCEST‐generated scenario‐specific acquisition schedules (Figure 3 and Supporting Information Table S1). Single‐shot QUESP‐EPI images were acquired with saturation at ±1× the chemical shift of each phantom’s exchangeable proton, except for the BSA where the existence of both the amide and rNOE pools is incompatible with QUESP estimation of the exchange rate. The QUESP saturation pulse powers ranged from 0 to 6 μT in 1 μT increments, the saturation pulse length ($T_{\mathrm{sat}}$) was 3 s, flip angle (FA) = $90^{\circ }$, and echo/repetition times (TE/TR) = 20/15000 ms. For comparison, a CEST‐MRF scan was performed, using a previously reported phantom acquisition schedule (Supporting Information Figure S1),^22^ shortened to include only the first N=10 images, for proper comparison with AutoCEST schedules of the same length. The CEST‐MRF protocol included a single saturation frequency offset (aimed at the target compound chemical shift frequency), TE/TR = 20/4000 ms, $T_{\mathrm{sat}}$ = 3 s, and FA = $60^{\circ }$. A traditional Z‐spectra was obtained using a CEST‐EPI protocol, employing a saturation pulse power of 2 μT, $T_{\mathrm{sat}}$ = 3 s, TE/TR = 20/8000 ms, and saturation frequency offsets of 7 to −7 ppm with 0.25 ppm increments. For calculation of the static magnetic field $B_{0}$ map using the water saturation shift referencing (WASSR) method,^42^ the CEST scan was repeated with a saturation pulse power of 0.3 μT, and frequency offsets ranging between 1 to −1 ppm with 0.1 ppm increments. $T_{1}$ maps were acquired using the variable repetition‐time rapid acquisition with relaxation enhancement (RARE) protocol, with TR = 50, 200, 400, 800, 1500, 3000, 5000, and 7500 ms, TE = 7.2 ms, RARE factor = 2. $T_{2}$ maps were acquired using the multi‐echo spin‐echo protocol, TR = 2000 ms, and 25 TE values between 20 and 500 ms.

#### 2.4.2 In vivo study

A quadrature volume coil was used for RF transmission and a mouse brain phased array surface coil was used for receive (Bruker Biospin, Billerica, MA). A field of view (FOV) of 19 × 19 mm^2^, a matrix of 64 × 64 pixels, and a 1 mm slice thickness were used in all scans except for a high‐resolution $T_{2}$‐weighted scan, where the matrix size was set to 128 × 128, and the TE/TR were 30/2000 ms. MT and amide AutoCEST scans were performed using the generated acquisition schedules described in Figure 3 and Supporting Information Table S1, with an echo time of 21.88 ms.

### 2.5 Comparison of different performance optimization methods

The suggested AutoCEST approach constitutes a *unified* framework for simultaneous optimization of both the acquisition protocol and the biophysical parameter quantification. To better understand the individual contributions of each optimization component (or neural network) to the overall performance optimization, as well as any synergistic effects between the two, the AutoCEST performance was compared to that of systems where:

1. A traditional MR‐fingerprint dot‐product quantification was applied on the data acquired using the *optimized* AutoCEST protocols.
2. A deep learning optimization of parameter quantification (similarly to the process described in Section 2.1.3) was applied on the data acquired using an *unoptimized* acquisition schedule.

The resulting images were compared to the images obtained from the *full* AutoCEST pipeline, as well as to those obtained from applying an *entirely* traditional MRF method using an unoptimized acquisition schedule (as described in Section 2.4.1 and Supporting Information Figure S1). The performance optimization method comparison was carried out for all in vitro and in vivo data obtained throughout this work.

### 2.6 Data analysis

Raw AutoCEST‐generated images were given as input to the trained reconstruction network, yielding the corresponding proton exchange rate and volume fraction maps. $T_{1}$ and $T_{2}$ exponential fitting were performed using a custom‐written program. Conventional CEST images were corrected for $B_{0}$ inhomogeneity using the WASSR method.^42^, ^43^ The ${\text{MTR}}_{\mathrm{asym}}$ was calculated using: ${\text{MTR}}_{\mathrm{asym}}=\left(S^{‐\Delta \omega }‐S^{+\Delta \omega }\right)/S_{0}$, where $S^{\pm \Delta \omega }$ is the signal measured with saturation at ± the relevant solute chemical shift and $S_{0}$ is the unsaturated signal. Exchange rate ground‐truth estimation was performed by fitting the QUESP data with the known solute concentration and measured water $T_{1}$ given as fixed inputs for each phantom vial.^44^ In addition, simultaneous QUESP estimation of both the exchange rate and the unconstrained solute concentration was performed for comparison.

CEST‐MRF signal matching was performed by calculating and finding the maximum dot‐product (after two‐norm normalization) of each pixel’s trajectory with all relevant simulated dictionary entries. The dictionaries were built using the same data properties used for training AutoCEST (Supporting Information Table S1). Dictionary generation was performed using a numerical solution of the Bloch–McConnell equations, implemented in MATLAB R2018a (The MathWorks, Natick, MA).^22^

In vitro statistics were calculated using 79 mm^2^ circular regions of interest (ROIs) drawn on each phantom vial. In vivo statistics were calculated using a gray matter (GM) ROI positioned on the cortex and a white matter (WM) ROI comprised of the corpus callosum and fiber tracts (cerebral peduncle, optic tract, and fimbria) regions. Localization of mouse brain regions was performed using the Allen Mouse Brain Atlas (adult mouse P56, coronal, image 78) as a reference.^45^, ^46^ Pearson’s correlation coefficients were calculated using the open source SciPy scientific computing library for Python.^47^ Absolute error was defined as |ground truth value − estimated value|. One‐way analysis of variance (ANOVA) followed by Tukey’s HSD test for comparing differences between multiple groups was performed using the Python module statsmodels.^48^ Differences were considered significant at p<0.05.

## 3 RESULTS

### 3.1 AutoCEST‐generated acquisition protocols

The AutoCEST optimization of a quantitative acquisition protocol took between 22 min and 5.58 hr (see Supporting Information Table S1). The optimized protocol acquisition time was 71.1 s for pCr, 47.6 s for L‐arginine, and 35s for all others (iohexol, BSA amide, BSA amine, BSA rNOE, in vivo amide, and in vivo MT). The optimized protocol parameters are shown in Figure 3.

### 3.2 Phantom study—exchange parameter quantification performance

**FIGURE 4:**
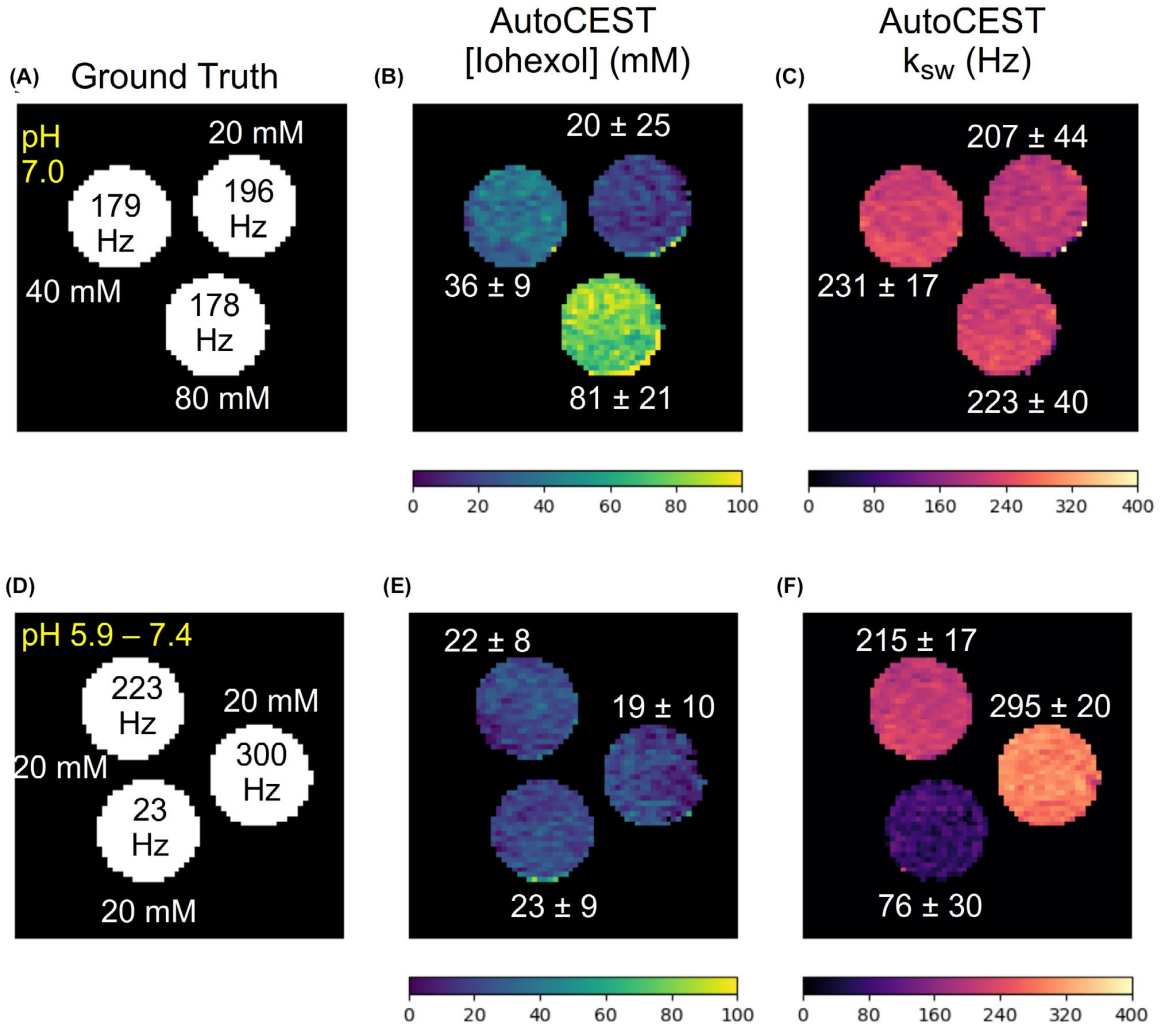
Iohexol phantom study. Each row represents a single phantom composed of three iohexol vials, with different concentrations (A) or pH (D). (B, E) AutoCEST‐generated iohexol concentration maps. (C, F) AutoCEST‐generated amide (4.3 ppm) proton exchange rate maps. The white text next to each vial represent its mean ± SD parameter value

**FIGURE 5:**
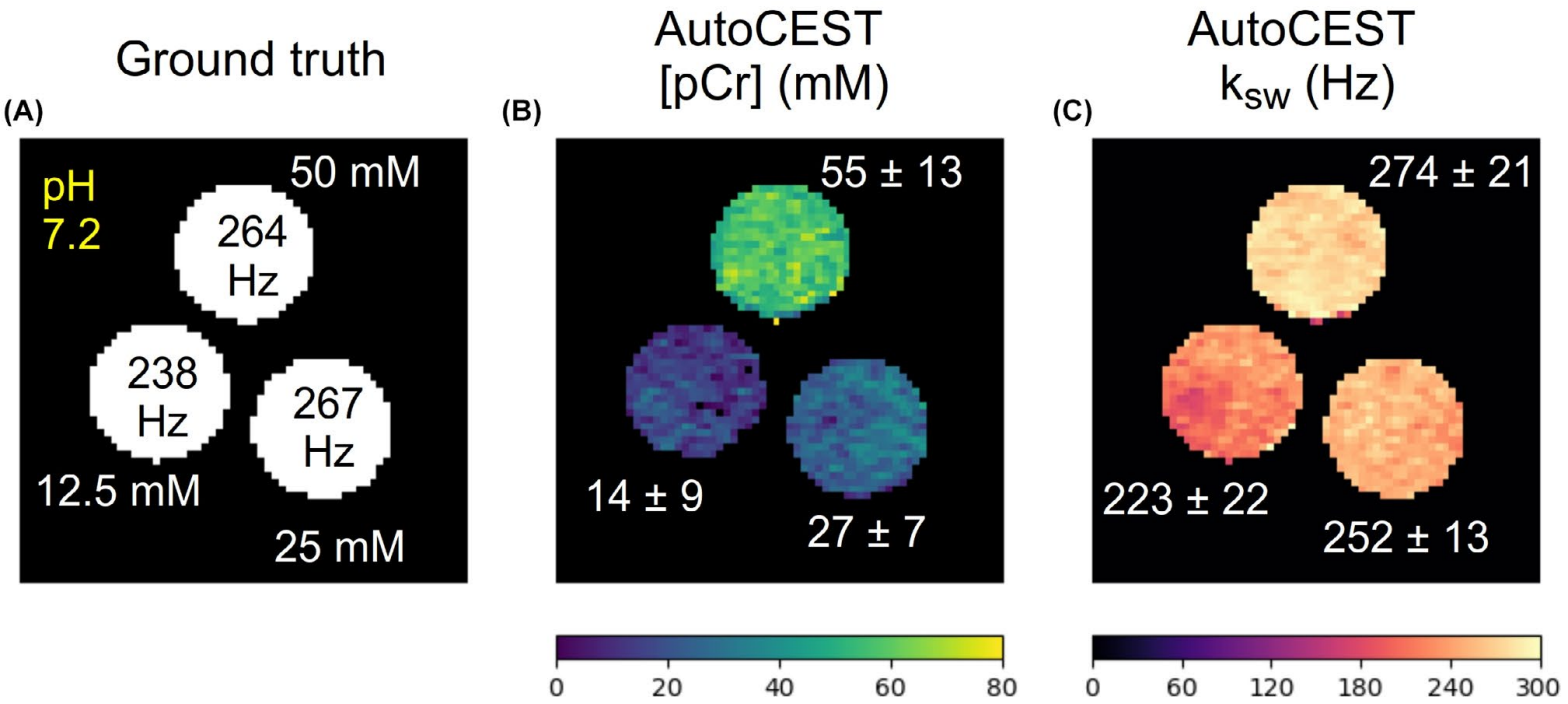
Phosphocreatine (pCr) phantom study. A, Ground truth solute concentration and pH. B, AutoCEST‐generated pCr concentration map. (C) AutoCEST‐generated guanidinium (2.6 ppm) proton exchange rate map. The white text next to each vial represent its mean ± SD parameter value

**FIGURE 6:**
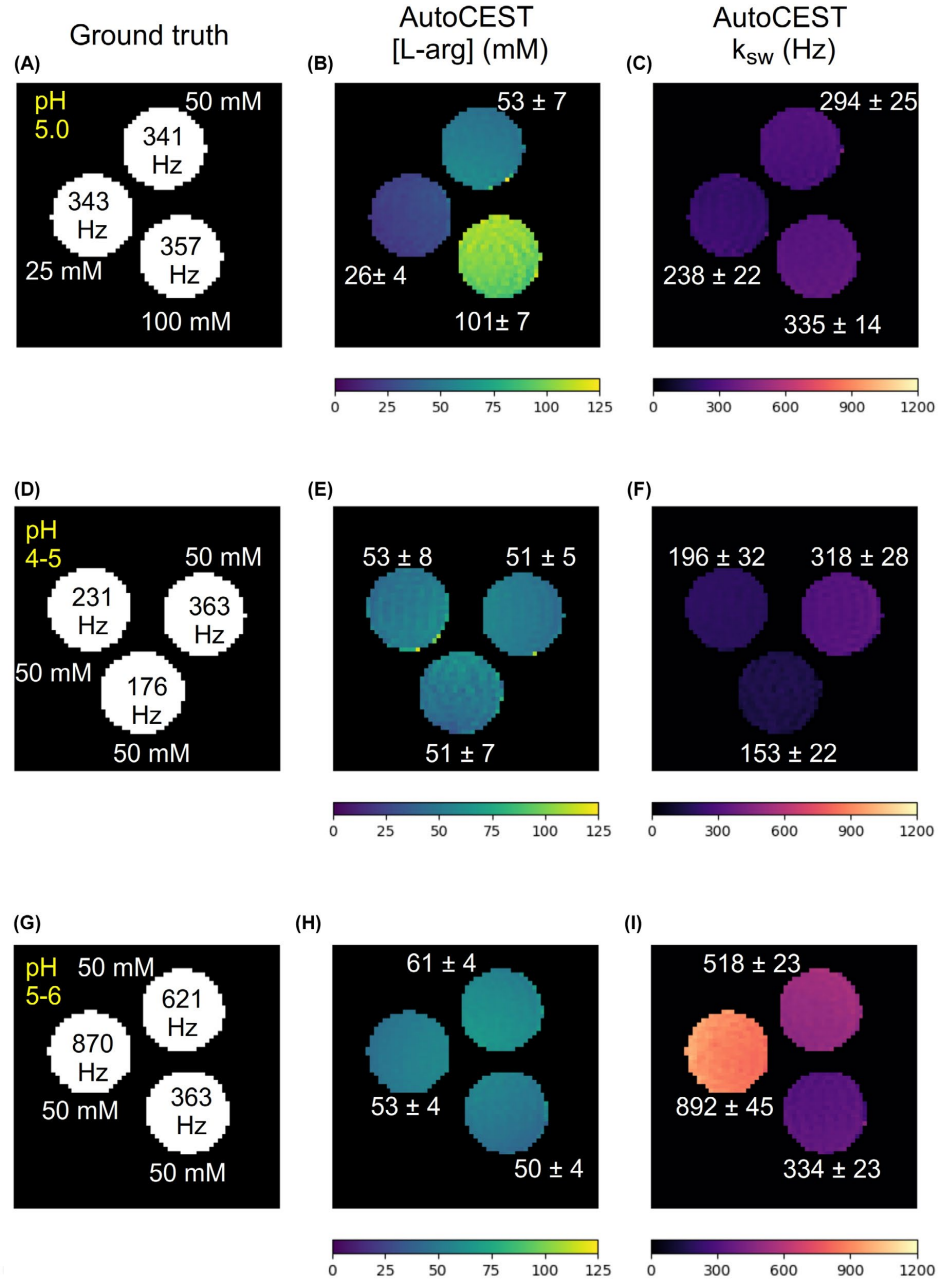
L‐arginine phantom study. Each row represents a single phantom composed of three L‐arginine vials, with different concentrations (A) or pH (D, G). (B, E, H) AutoCEST‐generated L‐arginine concentration maps. (C, F, I) AutoCEST‐generated amine (3 ppm) proton exchange rate maps. The white text next to each vial represent its mean ± SD parameter value

The AutoCEST reconstruction time for each pair of quantitative proton exchange rate and volume fraction maps (in vitro and in vivo) was 28.62 ± 0.01 ms. The resulting maps for iohexol, pCr, and L‐arg are shown in Figures 4–6, respectively. In all cases, an excellent agreement was oberved between the AutoCEST‐based calculated solute concentrations and the known solute concentrations, yielding an absolute error of 2.42 ± 2.53 mM and a significant correlation (Pearson’s r=0.992, p<0.0001). There was also a significant correlation between the QUESP‐calculated and AutoCEST‐measured proton exchange rates (r=0.971, p<0.0001), with an absolute error of 35.8 ± 29.3 Hz (Supporting Information Table S2).

The measured solute concentrations obtained with a pseudo‐random, unoptimized CEST‐MRF acquisition schedule (Supporting Information Figures S4–S9, panel E) were poorly correlated with the known solute concentrations (Pearson’s r=‐0.161, p=0.522), yielding an absolute error of 65.19 ± 34.48 mM. The absolute error between the QUESP‐calculated and CEST‐MRF measured proton exchange rates (Supporting Information Figures S4–S9, panel I) was higher than that obtained using AutoCEST (58.2 ± 56.76 Hz), yet there was a significant correlation between unoptimized CEST‐MRF and QUESP measured exchange rates (r=0.959, p<0.0001). The implementation of QUESP for simultaneous estimation of the concentration and exchange rate yielded a higher absolute error in solute concentration estimation compared to AutoCEST (11.03 ± 7.77 mM), and lower absolute error in proton exchange rate estimation (23.94 ± 29.54 Hz).

**FIGURE 7:**
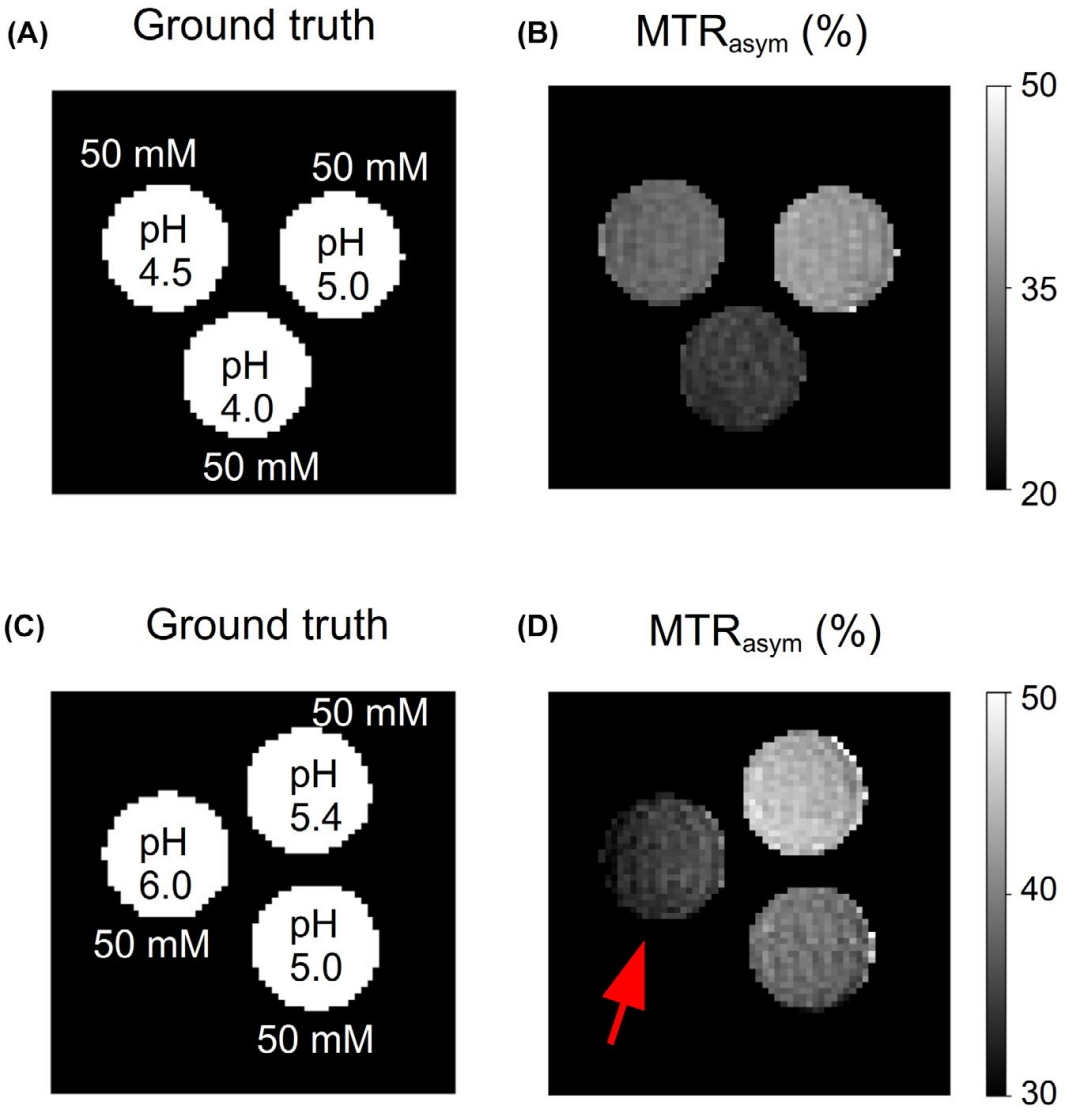
Conventional CEST‐weighted imaging. Each row represents a single phantom, composed of three L‐arginine vials, with different pH. (B, D) ${\text{MTR}}_{\mathrm{asym}}$ images obtained from a Z‐spectrum acquisition with a fixed saturation pulse power of 2 μT. The red arrow in D points to the highest pH vial, which demonstrated a decreased ${\text{MTR}}_{\mathrm{asym}}$ contrast due to insufficient saturation. AutoCEST‐generated maps of the same phantoms are available in Figure 6E,F,H,I

To demonstrate the differences between CEST‐weighted and AutoCEST output images, conventional ${\text{MTR}}_{\mathrm{asym}}$ images (acquired using a fixed saturation pulse power of 2 μT) for two L‐arginine phantoms are provided in Figure 7. Although the ${\text{MTR}}_{\mathrm{asym}}$ image in Figure 7B provides a clear contrast difference for different L‐arg vials, it cannot provide any definite information on the underlying biophysical mechanism; namely, whether a change in the solute concentration or pH is occurring. Moreover, the use of a single pulse saturation power is sub‐optimal for imaging scenarios with a wide possible range of proton exchange rates (or pH). This is demonstrated in Figure 7D, where an L‐arginine vial with fast exchanging protons (pH = 6) appears to have a decreased contrast, due to insufficient saturation. In contrast, a single AutoCEST imaging protocol was capable of correctly quantifying the exchange parameters and uncovering the chemical exchange property responsible for the change in contrast (Figure 6D–I).

**FIGURE 8:**
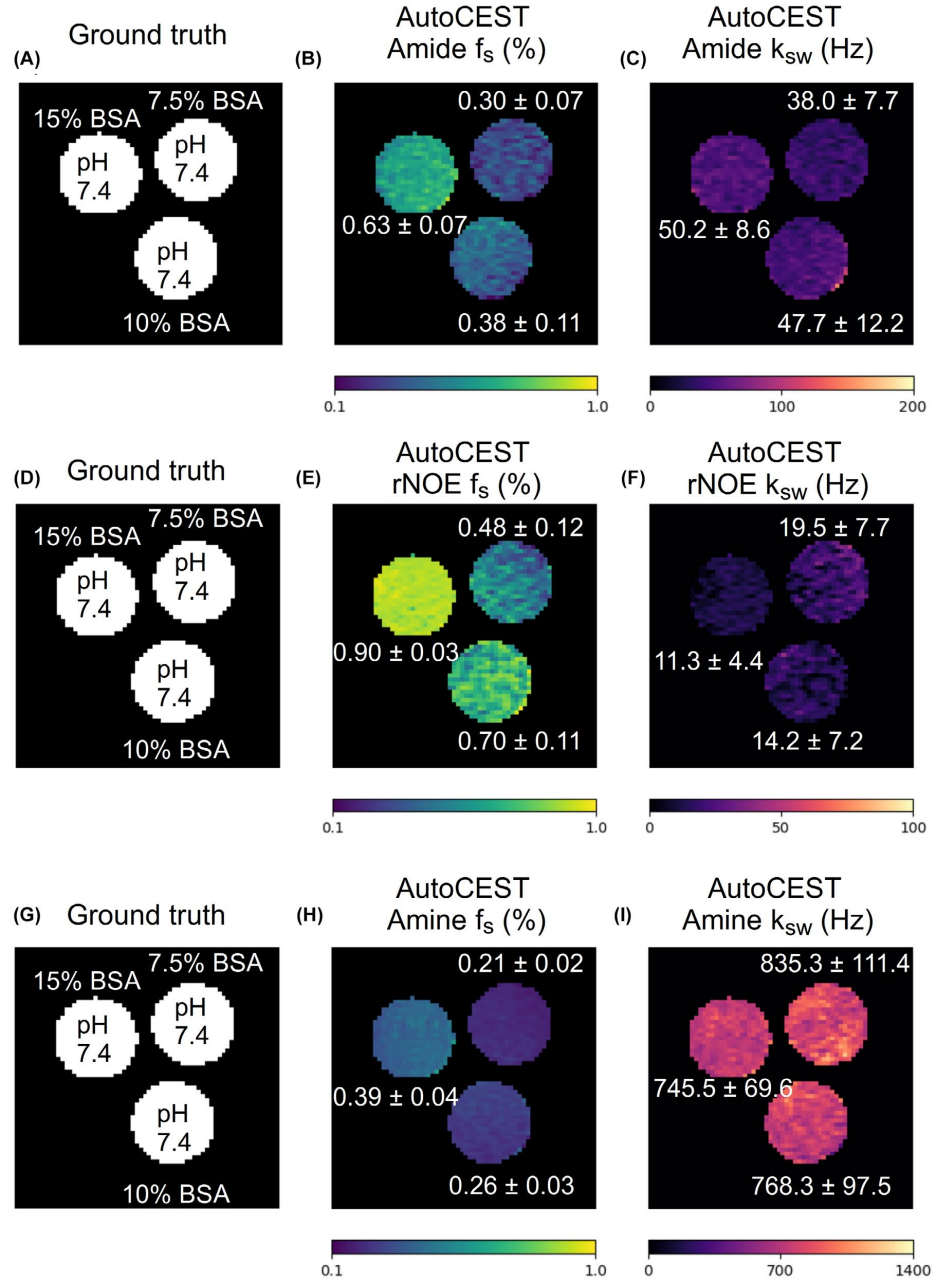
BSA phantom study. Each row represents a different molecular target (amide at 3.5 ppm, rNOE at −3.5 ppm, or amine at 2.75 ppm, respectively), imaged from the same phantom (A, D, G). (B, E, H) AutoCEST‐generated amide, rNOE, and amine proton volume fraction maps, respectively. (C, F, I) AutoCEST‐generated amide, rNOE, and amine proton exchange rate maps, respectively. The white text next to each vial represent its mean ± SD parameter value

AutoCEST quantitative images for the amide, rNOE, and amine exchangeable protons of BSA are shown in Figure 8. The proton volume fraction maps were in good agreement with the ground truth BSA concentration. AutoCEST‐based estimation of the exchange rates yielded parameter values (BSA amide ∼45 Hz, BSA rNOE ∼15 Hz, BSA amine ∼783 Hz) in good agreement with previous literature reports.^22^, ^41^, ^49^, ^50^

### 3.3 AutoCEST of in vivo mouse brain

**FIGURE 9:**
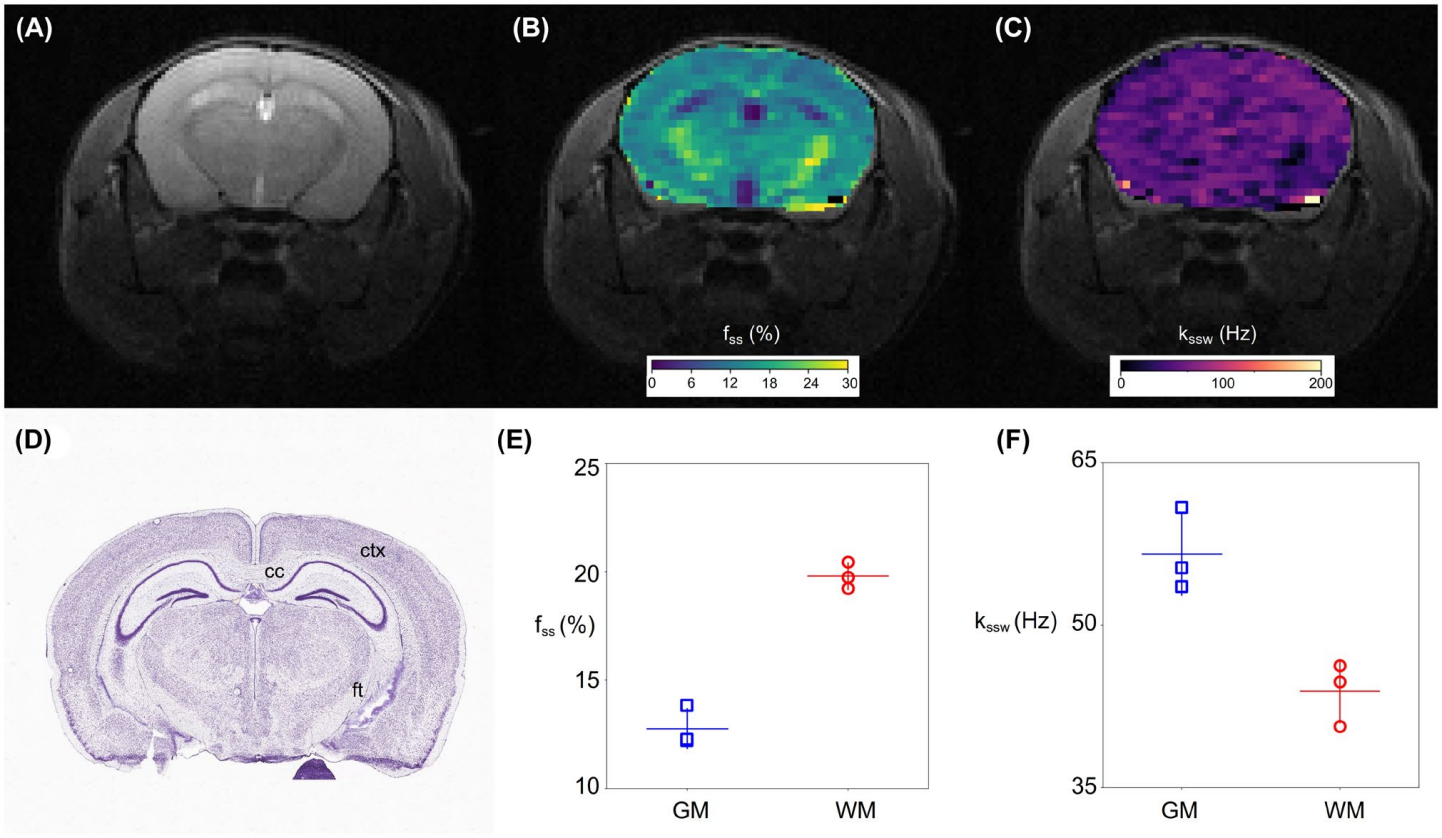
AutoCEST imaging of a representative in vivo mouse brain. (A) $T_{2}$‐weighted image and (D) corresponding Nissl‐stained mouse brain section with the cerebral cortex (ctx), corpus callosum (cc), and fiber tracts (ft, composed of cerebral peduncle, optic tract, and fimbria) identified.^45^, ^46^ AutoCEST‐generated (B) semi‐solid proton volume fraction ($f_{\mathrm{ss}}$) and (C) chemical exchange rate ($k_{\mathrm{ssw}}$) maps. (E, F). Analysis of the resulting exchange parameters in the white matter (WM, defined as the corpus callosum and white matter fiber tracts) and gray matter (GM). Data are presented as mean ± SD with all data points overlaid

Representative AutoCEST‐generated quantitative semi‐solid exchange parameter maps are shown in Figure 9, and additional results obtained for all mice are available in Supporting Information Figure S2 and Table S3. The semi‐solid proton volume fraction maps were in good agreement with the Nissl‐stained histology tissue section (Figure 9D), where neuronal cell bodies of GM are preferentially stained. In particular, an elevated semi‐solid volume fraction was observed for the subcortical WM (19.80% ± 0.50%) compared to the GM (12.77% ± 0.75%), allowing a clear identification of the corpus callosum and white matter fiber tracts. The obtained values were in good agreement with previous literature reports.^51^, ^52^ The semi‐solid chemical exchange rate was faster in the GM (56.54 ± 3.1 Hz) compared to WM (43.87 ± 2.36 Hz), in agreement with the literature.^24^, ^52^, ^53^

Amide exchange parameter maps for the same mouse used in Figure 9 are shown in Supporting Information Figure S3. The corresponding GM/WM parameter values are shown in Supporting Information Table S4. The AutoCEST‐generated amide proton volume fractions were 0.29% ± 0.16% and 0.40% ± 0.27% for the GM and WM, respectively. The amide proton exchange rates were 60.81 ± 9.28 Hz and 73.02 ± 51.11 Hz, for the GM and WM, respectively, which are in the general range of previously reported values,^24^, ^41^, ^54^ yet higher than the exchange rate measured using water exchange spectroscopy (WEX) in the rat cortex.^49^

### 3.4 Comparison of different performance optimization methods

**FIGURE 10:**
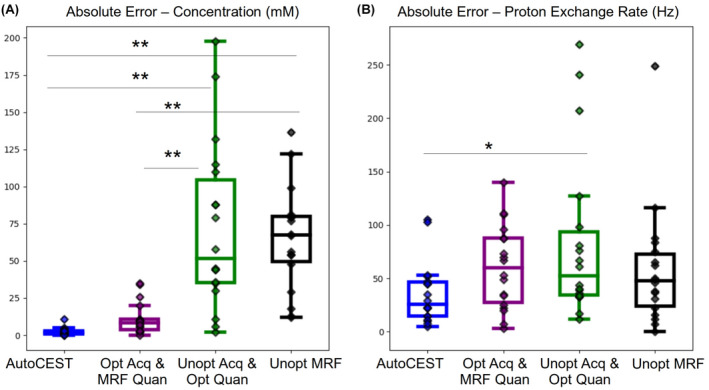
Absolute error analysis for the different optimization methods, based on the phantoms described in Figures 4, 5, and 6. A, Absolute error for compound concentration mapping. B, Absolute error for proton chemical exchange rate mapping. The evaluated methods were (left to right) AutoCEST (blue), dot‐product MRF quantification applied to data acquired using AutoCEST‐optimized schedules (purple), deep learning‐based quantification applied to data acquired using an unoptimized CEST‐MRF acquisition protocol (green), and CEST‐MRF dot‐product quantification applied to an unoptimized acquisition schedule (black). Statistical analysis of the resulting exchange parameters was carried out using one‐way analysis of variance (ANOVA) followed by Tukey’s HSD test (n=18 phantom vials). *p<0.05; **p<0.01. In all box plots, the central horizontal lines represent median values, box limits represent upper (third) and lower (first) quartiles, whiskers represent 1.5× the interquartile range above and below the upper and lower quartiles, respectively, and all data points are plotted

The performance of the full AutoCEST pipeline was compared to that of individual elements of the pipeline to examine the importance of each element to the overall performance optimization (Supplementary Information Figures S4–S16). A statistical analysis comparing the different optimization variants is provided in Figure 10, where the absolute error for each case was calculated using the phantoms where the most reliable ground truth was available (measured concentration and QUESP‐derived proton exchange rate). The best performance was observed for the full AutoCEST pipeline, where a sigificantly lower absolute error in quantifying the compound concentration (p<0.01, n=18 phantom vials, one‐way ANOVA followed by Tukey’s HSD test) was obtained compared to the use of unoptimized acquisition schedules (with or without deep NN quantification). Nevertheless, using the AutoCEST‐dervied acquisition schedules for “classical” dot‐prodcut MRF quantification yielded significantly lower errors (p<0.01, n=18 phantom vials, one‐way ANOVA followed by Tukey’s HSD test) compared to unoptimized MRF acquisition, demonstrating the potential of AutoCEST for also serving as a means for CEST‐MRF protocol optimization. Notably, the chemical exchange rate is generally more challenging for quantification compared to the compound concentration. It is therefore not surprising that the differences in the exchange rate errors between the different optimization methods were less striking than for the concentration. Nevertheless, the median absolute error and the standard deviations in the quantification error were much smaller for AutoCEST compared to other methods.

## 4 DISCUSSION

Since its establishment more than 20 years ago, CEST MRI has been increasingly investigated as a promising contrast mechanism for studying a variety of disease pathologies. However, while numerous clinical CEST studies have demonstrated its potential,^2^ this technique has not yet been adopted in routine clinical practice. The main barriers for clinical translation have been the typically long image acquisition times, the semi‐quantitative nature of the proton exchange‐weighted image contrast, which depends on a complex overlay of contrasts from different exchangeable proton pools (MT, rNOE, amide, amine), and the inability to separate out contributions to the CEST contrast from chemical exchange rate and proton volume fraction, both of which may be changing with time and disease progression. A quantitative and rapid imaging approach could drastically improve the clinical applicability of CEST, rendering it as an attractive means for gaining new diagnostic insights.

A CEST MRF approach could help overcome the above challenges and provide quantitative CEST and MT information.^22^, ^23^, ^24^ Recently, it was further combined with deep learning architectures, for rapid MT^55^, ^56^ and CEST/MT^41^ fingerprinting. However, previous studies have also demonstrated that the ability to discriminate different exchange parameter values depends critically on the choice of acquisition schedule.^22^, ^25^ In particular, the transfer of a CEST‐MRF acquisition protocol from one chemical exchange scenario to another is not straight‐forward,^25^ requiring a through optimization, validation with appropriate tissue‐like phantoms, and expert knowledge of the effect of the acquisition protocol properties on the resulting CEST signals. As demonstrated here, naively taking a random CEST‐MRF acquisition schedule, which might be useful for a particular CEST agent and applying it for other compounds/applications, could result in very poor performance. This is demonstrated in Supporting Information Figures S4–S12 panels E and I, where poor agreement is observed between the exchange parameters determined from an unoptimized CEST‐MRF acquisition schedule and the known ground truth values for Iohexol (Supporting Information Figures S4–5E,I), phosphocreatine (Supporting Information Figure S6E,I), L‐arginine (Supporting Information Figures S7–9E,I), and BSA (Supporting Information Figures S10‐12E,I) phantoms. In contrast, here we demonstrate that AutoCEST can adapt and optimize the acquisition schedule for a variety of distinctly different chemical exchange scenarios, accurately mapping the exchange parameters (Figures 4–10, Supporting Information Table S2). In addition, AutoCEST was able to accurately map the solute concentration and chemical exchange rate in a very short time with acquisition times of only 35–71 s and an almost instantaneous reconstruction time of 29 ms. This dramatically reduced scan time could greatly assist in incorporating CEST investigations into routine clinical imaging with minimal interference with workflow or time constraints.

The AutoCEST method proposed here constitutes a unified framework for both the design of fast CEST/MT acquisition protocols and the reconstruction of quantitative parameter maps. Importantly, the method is fully automatic, removing the need for user‐dependent analysis and exhaustive tuning and optimization of acquisition protocols. The AutoCEST realization was inspired and driven by the AutoSeq method, which allows for automatic sequence generation in 1D and single pixel $T_{1}$/$T_{2}$ quantitative imaging.^28^, ^57^ Recently, the MRzero^58^ method was reported, which further incorporates gradient and RF‐events for learning 2D imaging acquisition schedules, including free k‐space trajectories.^59^ The present work expands on the idea of AI‐based sequence design for CEST/MT quantitative imaging, where a crucial need for automatic schedule invention lies. Observing the differences between the acquisition schedules used for AutoCEST initialization and the final optimized schedules (Figure 3), can provide some intuition into the underlying optimization performed. For example, optimization of the acquisition schedules for both the Iohexol (at room temperature) and BSA‐amide imaging scenarios resulted in saturation pulse powers that were lower than initialized. This can be explained by the relatively slow exchange rates of these compounds (<300 Hz) which are not expected to benefit from a high saturation power. Similarly, the optimal saturation frequency offset for amide and amine exchangeable protons remained roughly fixed at the solute frequency offset, as expected for a CEST agent with a relatively narrow spectral width (Figure 3O), while the spectrally very broad semi‐solid MT case required a wider range of saturation pulse frequency offsets (Figure 3G).

The particular patterns obtained for some of the optimized parameters appeared to lack any noticeable human‐intuition (Figure 3E–J), similar to the results obtained in $T_{1}$/$T_{2}$ MRF sequence generation.^60^ This highlights the need for an automated computer‐based optimization process. In addition, although the resulting optimized protocols were mostly substantially different than the initial acquisition schedules, there were a few cases where the protocols were not drastically modified (Figure 3C,D). This might explain the success of some previously reported random CEST‐MRF schedules, which could in some cases randomly “land on” suitable parameters.

The AutoCEST‐generated schedules tended to have a longer recovery time compared to the initial value. Notably, quantitative CEST is characterized by an internal trade‐off between a sufficiently high SNR and a clinically relevant scan time.^25^ While longer recovery times improve the former, some compromise must be made to accommodate for the latter. In this work, we have either fixed or limited the lower and upper bounds for the AutoCEST optimized $T_{\mathrm{sat}}$ and $T_{\mathrm{rec}}$ (Supporting Information Table S1). Although probably not reaching the optimal possible sensitivity, this approach has yielded very good performance (Supporting Information Table S2), while satisfying the need for a short scan time with all output schedules shorter than 72 s.

All the experiments conducted in this work were fixed to create acquisition schedules of N=10 raw images, together with additional restrictions on the scan time (in the form of maximal $T_{\mathrm{rec}}$ and $T_{\mathrm{sat}}$, Supporting Information Table S1). While this was done to push the boundaries of quantitative CEST beyond the limits set by previous work, a slight relaxation in the parameter restriction could improve the quantitation performance, and still retain sufficiently clinically relevant scan times. In the future, the number of raw images acquired (N) could be defined as a dynamically optimized parameter. In addition, while the saturation power was limited to not exceed a fixed value for each of the scenarios (Supporting Information Table S1), it could be replaced in the future by a specific absorption rate (SAR) penalty term, incorporated in the cost‐function.^58^ Similarly, a penalty term for exceedingly long scan times could be used to further improve SNR/scan‐time balance.

The AutoCEST determined exchange parameters for the in vivo mouse brain were in general agreement with the literature for two‐pool MT and three‐pool amide/MT imaging; however, the resulting in vivo amide exchange rates were higher than a previous WEX estimation in the rat cortex^49^ and demonstrated a rather large standard deviation (Supporting Information Table S4). Although amide chemical exchange rate is a subject of some controversy in the field, given that various groups have reported amide proton exchange rates >100 Hz,^24^, ^61^ it might be useful to pursue additional strategies for exploring multi‐pool AutoCEST imaging. In particular, the use of a single acquisition schedule, with saturation at the amide proton frequency only, may make discrimination of both amide and MT pool exchange parameters more challenging. For example, we have recently demonstrated that nailing down the MT pool parameters, with an MT‐specific acquisition schedule, and then sequentially using them as direct inputs for the amide‐pool classification, significantly improved the performance in CEST‐MRF of oncolytic virotherapy treated mice.^41^ Future work could expand the architecture of AutoCEST to allow for such sequentially acquired information to be incorporated. In addition, several other compounds could be added to the model and simulations for improved accuracy, such as glutamate and guanidyl amine protons.

While the experiments described here were all performed on preclinical scanners with continuous wave saturation pulses, the implementation of AutoCEST for clinical scanners could be straightforwardly translated for cases in which a single continuous‐wave block pulse could be applied (e.g., when the required $T_{\mathrm{sat}}$ and/or $B_{1\max}$ are not expected to be too large), or by modifying the analytical solution of the CEST saturation block to accommodate for a pulse train.^62^, ^63^, ^64^

## 5 CONCLUSION

The suggested framework provides a fast and automatic means for designing and analyzing quantitative CEST experiments, potentially contributing to the efforts to disseminate CEST/MT in the clinic. The superiority of AutoCEST performance compared to unoptimized CEST MRF highlights the importance of optimizing the acquisition schedule for improved discrimination of the exchange parameters.

## Supporting information


**TABLE S1** Detailed properties of the simulated data used for training AutoCEST
**TABLE S2** Comparison of the concentrations and proton chemical exchange rates determined by AutoCEST, CEST‐MRF, and QUESP
**TABLE S3** AutoCEST‐determined semi‐solid proton chemical exchange rates ($k_{\mathrm{ssw}}$) and volume fractions ($f_{\mathrm{ss}}$) for GM and WM brain tissue regions from three in vivo mice
**TABLE S4** AutoCEST‐determined amide proton chemical exchange rates ($k_{\mathrm{sw}}$) and volume fractions ($f_{s}$) for GM and WM brain tissue regions from an in vivo mouse
**FIGURE S1** A previously reported phantom acquisition schedule,^22^ shortened to N=10 images and used as a reference unoptimized CEST‐MRF protocol. The saturation pulse duration was 3 s, the recovery time was 1s, the readout flip angle was $60^{\circ }$, and the saturation pulse frequency was set to the chemical shift of the exchangeable proton of each imaged phantom
**FIGURE S2** AutoCEST brain imaging of three in vivo mice. Each row represents a different animal with T2 ‐weighted images (A, D, G) and AutoCEST‐generated semi‐solid proton volume fraction (B, E, H) and chemical exchange rate maps (C, F, I)
**FIGURE S3** AutoCEST amide proton exchange parameter mapping of an in vivo mouse. A, T2‐weighted image. B, AutoCEST‐generated amide proton volume fraction (fs). C, AutoCEST‐generated amide proton chemical exchange rate (ksw)
**FIGURE S4** Comparison of different performance optimization methods—iohexol phantom with various concentrations. A, Ground truth concentrations and QUESP‐determined proton exchange rates. The top row shows the resulting Iohexol concentration maps and the bottom row shows the resulting amide (4.3 ppm) proton exchange rate maps obtained using (B, F) AutoCEST, (C, G) dot‐product MRF quantification applied to data acquired using AutoCEST‐optimized schedules, (D, H) deep learning‐based quantification applied to data acquired using an unoptimized CEST‐MRF acquisition protocol, and (E, I) CEST‐MRF dot‐product quantification applied to an unoptimized acquisition schedule. The white text next to each vial represent its mean ± SD parameter value
**FIGURE S5** Comparison of different performance optimization methods—Iohexol phantom with various pH levels. A, Ground truth concentrations and QUESP‐determined proton exchange rates. The top row shows the resulting Iohexol concentration maps and the bottom row shows the resulting amide (4.3 ppm) proton exchange rate maps obtained using (B, F) AutoCEST, (C, G) dot‐product MRF quantification applied to data acquired using AutoCEST‐optimized schedules, (D, H) deep learning‐based quantification applied to data acquired using an unoptimized CEST‐MRF acquisition protocol, and (E, I) CEST‐MRF dot‐product quantification applied to an unoptimized acquisition schedule. The white text next to each vial represent its mean ± SD parameter value
**FIGURE S6** Comparison of different performance optimization methods—Phosphocreatine (pCr) phantom. A, Ground truth concentrations and QUESP‐determined proton exchange rates. The top row shows the resulting pCr concentration maps and the bottom row shows the resulting guanidinium (2.6 ppm) proton exchange rate maps obtained using (B, F) AutoCEST, (C, G) dot‐product MRF quantification applied to data acquired using AutoCEST‐optimized schedules, (D, H) deep learning‐based quantification applied to data acquired using an unoptimized CEST‐MRF acquisition protocol, and (E, I) CEST‐MRF dot‐product quantification applied to an unoptimized acquisition schedule. The white text next to each vial represent its mean ± SD parameter value
**FIGURE S7** Comparison of different performance optimization methods—L‐arginine phantom with various concentrations. A, Ground truth concentrations and QUESP‐determined proton exchange rates. The top row shows the resulting L‐arginine concentration maps and the bottom row shows the resulting amine (3 ppm) proton exchange rate maps obtained using (B, F) AutoCEST, (C, G) dot‐product MRF quantification applied to data acquired using AutoCEST‐optimized schedules, (D, H) deep learning‐based quantification applied to data acquired using an unoptimized CEST‐MRF acquisition protocol, and (E, I) CEST‐MRF dot‐product quantification applied to an unoptimized acquisition schedule. The white text next to each vial represent its mean ± SD parameter value
**FIGURE S8** Comparison of different performance optimization methods—L‐arginine phantom with pH 4–5. A, Ground truth concentrations and QUESP‐determined proton exchange rates. The top row shows the resulting L‐arginine concentration maps and the bottom row shows the resulting amine (3 ppm) proton exchange rate maps obtained using (B, F) AutoCEST, (C, G) dot‐product MRF quantification applied to data acquired using AutoCEST‐optimized schedules, (D, H) deep learning‐based quantification applied to data acquired using an unoptimized CEST‐MRF acquisition protocol, and (E, I) CEST‐MRF dot‐product quantification applied to an unoptimized acquisition schedule. The white text next to each vial represent its mean ± SD parameter value
**FIGURE S9** Comparison of different performance optimization methods—L‐arginine phantom with pH 5–6. A, Ground truth concentrations and QUESP‐determined proton exchange rates. The top row shows the resulting L‐arginine concentration maps and the bottom row shows the resulting amine (3 ppm) proton exchange rate maps obtained using (B, F) AutoCEST, (C, G) dot‐product MRF quantification applied to data acquired using AutoCEST‐optimized schedules, (D, H) deep learning‐based quantification applied to data acquired using an unoptimized CEST‐MRF acquisition protocol, and (E, I) CEST‐MRF dot‐product quantification applied to an unoptimized acquisition schedule. The white text next to each vial represent its mean ± SD parameter value
**FIGURE S10** Comparison of different performance optimization methods—BSA phantom with amide (3.5 ppm) as the target compound. A, Ground truth BSA concentrations and pH. The top and bottom rows show the resulting amide proton volume fraction and exchange rate maps, respectively, obtained using (B, F) AutoCEST, (C, G) dot‐product MRF quantification applied to data acquired using AutoCEST‐optimized schedules, (D, H) deep learning‐based quantification applied to data acquired using an unoptimized CEST‐MRF acquisition protocol, and (E, I) CEST‐MRF dot‐product quantification applied to an unoptimized acquisition schedule. The white text next to each vial represent its mean ± SD parameter value
**FIGURE S11** Comparison of different performance optimization methods—BSA phantom with aliphatic rNOE (−3.5 ppm) as the target compound. A, Ground truth BSA concentrations and pH. The top and bottom rows show the resulting rNOE proton volume fraction and exchange rate maps, respectively, obtained using (B, F) AutoCEST, (C, G) dot‐product MRF quantification applied to data acquired using AutoCEST‐optimized schedules, (D, H) deep learning‐based quantification applied to data acquired using an unoptimized CEST‐MRF acquisition protocol, and (E, I) CEST‐MRF dot‐product quantification applied to an unoptimized acquisition schedule. The white text next to each vial represent its mean ± SD parameter value
**FIGURE S12** Comparison of different performance optimization methods—BSA phantom with amine proton (2 ppm) as the target compound. A, Ground truth BSA concentrations and pH. The top and bottom rows show the resulting amine proton volume fraction and exchange rate maps, respectively, obtained using (B, F) AutoCEST, (C, G) dot‐product MRF quantification applied to data acquired using AutoCEST‐optimized schedules, (D, H) deep learning‐based quantification applied to data acquired using an unoptimized CEST‐MRF acquisition protocol, and (E, I) CEST‐MRF dot‐product quantification applied to an unoptimized acquisition schedule. The white text next to each vial represent its mean ± SD parameter value
**FIGURE S13** Comparison of different performance optimization methods—in vivo MT imaging, animal #1. The top and bottom rows show the resulting semi‐solid proton volume fraction and chemical exchange rate maps, respectively, obtained using (A, E) AutoCEST, (B, F) dot‐product MRF quantification applied to data acquired using AutoCEST‐optimized schedules, (C, G) deep learning‐based quantification applied to data acquired using an unoptimized CEST‐MRF acquisition protocol, and (D, H) CEST‐MRF dot‐product quantification applied to an unoptimized acquisition schedule
**FIGURE S14** Comparison of different performance optimization methods—vivo MT imaging, animal #2. The top and bottom rows show the resulting semi‐solid proton volume fraction and chemical exchange rate maps, respectively, obtained using (A, E) AutoCEST, (B, F) dot‐product MRF quantification applied to data acquired using AutoCEST optimized schedules, (C, G) deep learning‐based quantification applied to data acquired using an unoptimized CEST‐MRF acquisition protocol, and (D, H) CEST‐MRF dot‐product quantification applied to an unoptimized acquisition schedule
**FIGURE S15** Comparison of different performance optimization methods—in vivo MT imaging, animal #3. The top and bottom rows show the resulting semi‐solid proton volume fraction and chemical exchange rate maps, respectively, obtained using (A, E) autoCEST, (B, F) dot‐product MRF quantification applied to data acquired using AutoCEST optimized schedules, (C, G) deep learning‐based quantification applied to data acquired using an unoptimized CEST‐MRF acquisition protocol, and (D, H) CEST‐MRF dot‐product quantification applied to an unoptimized acquisition schedule
**FIGURE S16** Comparison of different performance optimization methods—in vivo amide imaging. The top and bottom rows show the resulting amide proton volume fraction and chemical exchange rate maps, respectively, obtained using (A, E) autoCEST, (B, F) dot‐product MRF quantification applied to data acquired using AutoCEST‐optimized schedules, (C, G) deep learning‐based quantification applied to data acquired using an unoptimized CEST‐MRF acquisition protocol, and (D, H) CEST‐MRF dot‐product quantification applied to an unoptimized acquisition schedule

## Data Availability

The raw and analyzed AutoCEST data used in this work are available in https://doi.org/10.6084/m9.figshare.14877765. MR‐fingerprinting dictionaries can be reproduced using the open‐source code available in https://pulseq‐cest.github.io ^52^ with the parameters described in Supporting Information Table S1. Conventional CEST analysis can be performed using the code available in https://github.com/cest‐sources. Source code is available from the corresponding author upon request.
