# Supplementary material for: An end‐to‐end AI‐based framework for automated discovery of rapid CEST/MT MRI acquisition protocols and molecular parameter quantification (AutoCEST)
Source: Magn Reson Med. 2022 Jan 28;87(6):2792–810. doi: 10.1002/mrm.29173 (PMC9305180; doi:10.1002/mrm.29173)
Supplement: Supplementary file 1 — TABLE S1 Detailed properties of the simulated data used for training AutoCEST TABLE S2 Comparison of the concentrations and proton chemical exchange rates determined by AutoCEST, CEST‐MRF, and QUESP TABLE S3 AutoCEST‐determined semi‐solid proton chemical exchange rates (kssw) and volume fractions (fss) for GM and WM brain tissue regions from three in vivo mice TABLE S4 AutoCEST‐determined amide proton chemical exchange rates (ksw) and volume fractions (fs) for GM and WM brain tissue regions from an in vivo mouse FIGURE S1 A previously reported phantom acquisition schedule, 22 shortened to N=10 images and used as a reference unoptimized CEST‐MRF protocol. The saturation pulse duration was 3 s, the recovery time was 1s, the readout flip angle was 60∘, and the saturation pulse frequency was set to the chemical shift of the exchangeable proton of each imaged phantom FIGURE S2 AutoCEST brain imaging of three in vivo mice. Each row represents a different animal with T2 ‐weighted images (A, D, G) and AutoCEST‐generated semi‐solid proton volume fraction (B, E, H) and chemical exchange rate maps (C, F, I) FIGURE S3 AutoCEST amide proton exchange parameter mapping of an in vivo mouse. A, T2‐weighted image. B, AutoCEST‐generated amide proton volume fraction (fs). C, AutoCEST‐generated amide proton chemical exchange rate (ksw) FIGURE S4 Comparison of different performance optimization methods—iohexol phantom with various concentrations. A, Ground truth concentrations and QUESP‐determined proton exchange rates. The top row shows the resulting Iohexol concentration maps and the bottom row shows the resulting amide (4.3 ppm) proton exchange rate maps obtained using (B, F) AutoCEST, (C, G) dot‐product MRF quantification applied to data acquired using AutoCEST‐optimized schedules, (D, H) deep learning‐based quantification applied to data acquired using an unoptimized CEST‐MRF acquisition protocol, and (E, I) CEST‐MRF dot‐product quantification applied to an unoptimized acquisition s [file MRM-87-2792-s001.pdf]

Supporting Information Table S1. Detailed properties of the simulated data used for training AutoCEST.

Compound of interest	Iohexol	pCr	L-arginine	BSA - amide	BSA - amine	BSA - rNOE	In Vivo MT	In Vivo Amide***
Water T_1 (ms)	2100:50:2700*	3300:50:3800	2500:50:3300	2000:50:3400	2600:50:3400	2600:50:3400	1300:100:2600	1300:200:2500
Water T_2 (ms)	200:25:650	1000:50:1600	600:50:1200	50:10:300	50:10:300	50:10:300	40:10:130	40:20:120
Solute/semi-solid T_1 (ms)	2200	1000	2800	Equal to water T_1[65]	Equal to water T_1[65]	Equal to water T_1[65]	Equal to water T_1[65]	Equal to water T_1[65]
Solute/semi-solid T_2 (ms)	40	500[40]	40	1	1	0.5[66]	0.04**	1
Solute/semi-solid chemical shift (ppm)	4.3	2.6	3	3.5	2.75	-3.5	-2.5	3.5
Number of exchangeable protons	2	1	3	1	1	1	1	1
k_{sw} / k_{ssw} (Hz)	10:10:300	50:5:200	100:10:1400	5:5:100	50:50:1500	5:5:100	5:5:100	5:5:100
Solute/semi-solid concentration (mM)	10:5:100	2:2:150	10:5:120	100:50:1000	20:20:600	50:50:1000	2k:2k:30k	100:50:1000
Optimized parameters and constraints (if any)	$B_1 \leq 6$ μT	$B_1 \leq 1.5$ μT<br>$T_{sat} \leq 5$s<br>$T_{rec} \leq 5$s	$B_1 \leq 6$ μT<br>1.5s $\leq T_{sat} \leq$ 3s<br>1.5s $\leq T_{rec} \leq$ 2.5s<br>FA<br>ω_{rf}	$B_1 \leq 4$ μT	$B_1 \leq 6$ μT	$B_1 \leq 4$ μT	$B_1 \leq 4$ μT<br>10 ppm $\leq \omega_{rf} \leq$ 75 ppm	$B_1 \leq 2$ μT
Fixed acquisition parameters	T_{sat} = 2.5s<br>T_{rec} = 1s<br>ω_{rf} = 4.3 ppm<br>FA = 90°	ω_{rf} = 2.6 ppm<br>FA = 90°	none	T_{sat} = 2.5s<br>T_{rec} = 1s<br>ω_{rf} = 3.5 ppm<br>FA = 90°	T_{sat} = 2.5s<br>T_{rec} = 1s<br>ω_{rf} = 2.75 ppm<br>FA = 90°	T_{sat} = 2.5s<br>T_{rec} = 1s<br>ω_{rf} = -3.5 ppm<br>FA = 90°	T_{sat} = 2.5s<br>T_{rec} = 1s<br>FA = 90°	T_{sat} = 2.5s<br>T_{rec} = 1s<br>ω_{rf} = 3.5 ppm<br>FA = 90°
Total number of simulated signals	140,790	332,475	665,873	286,520	397,800	176,800	42,000	532,000
Time required for AutoCEST to generate the optimized acquisition schedule (using a Linux Laptop with 8 CPUs)	32 min	2.50 hrs	5.58 hrs	2.38 hrs	3.28 hrs	1.61 hrs	22 min	4.07 hrs

*The notation x:y:z represents a discrete range of values between [x, z] with y increments.

**The Bloch-McConnell equations-based data generator yielded a Lorentzian line-shape for the semi-solid pool. To generate a linewidth equivalent to the commonly reported super-Lorentzian of 10 μs[53], a four times higher value (40 μs) was input to the dictionary generator[65].

***A 3-pool scenario was simulated, by including both the amide and MT pool parameters, as described in the in-vivo MT column, with semi-solid concentrations and exchange rates of 2k:4k:30k and 10:20:90, respectively.

[65]Zaiß M, Schmitt B, Bachert P. Quantitative separation of CEST effect from magnetization transfer and spillover effects by Lorentzian-line-fit analysis of z-spectra. *J Magn Reson.* 2011;211:149-155.

[66]Zhang X-Y, Wang F, Li H, et al. Accuracy in the quantification of chemical exchange saturation transfer (CEST) and relayed nuclear Overhauser enhancement (rNOE) saturation transfer effects. *NMR Biomed.* 2017;30:e3716.

Supporting Information Table S2. **Comparison of the concentrations and proton chemical exchange rates determined by AutoCEST, CEST-MRF, and QUESP.**

Phantom	Ground truth		QUESP - ground truth concentration as input	QUESP - simultaneous estimation of concentration and exchange rate		AutoCEST		Unoptimized CEST-MRF	
	**Concentration (mM)**	**pH**	**k_{sw} (Hz)**	**Concentration (mM)**	**k_{sw} (Hz)**	**Concentration (mM)**	**k_{sw} (Hz)**	**Concentration (mM)**	**k_{sw} (Hz)**
pCr	50	7.2	264±158	27±26	257±156	55±13	274±21	149±9	199±11
	25	7.2	267±356	13±28	267±356	27±7	252±13	147±8	179±15
	12.5	7.2	238±650	8±45	193±610	14±9	223±22	149±4	154±16
Iohexol	20	7.0	196±24	10±5	271±108	20±25	207±44	97±10	134±30
	80	7.0	178±12	47±5	232±21	81±21	223±40	98±8	228±25
	40	7.0	179±14	21±3	241±32	36±9	231±17	94±13	172±27
	20	7.4	300±287	27±44	300±503	19±10	295±20	100±0	300±0
	20	5.9	23±2734	15±1271	29±2305	23±9	76±30	100±4	72±19
	20	6.8	223±24	10±4	294±87	22±8	215±17	99±6	148±25
L-arg	50	5.0	341±43	41±5	340±43	53±7	294±25	106 ± 15	303 ± 35
	100	5.0	357±28	93±10	349±49	101±7	335±14	112 ± 15	403 ± 33
	25	5.1	343±46	18±2	343±46	26± 4	238 ± 22	106 ± 15	227 ± 22
	50	5.0	363±42	43±4	365±42	51 ± 5	318 ± 28	104 ± 18	326 ± 38
	50	4.0	176±41	36±10	176±41	51 ± 7	153 ± 22	118 ± 8	154 ± 19
	50	4.5	231±39	36±7	232±39	53 ± 8	196 ± 32	117 ± 7	200 ± 20
	50	5.4	621±122	51±4	549±65	61 ± 4	518 ± 23	79 ± 15	605 ± 50
	50	5.0	363±20	37±4	378±44	50 ± 4	334 ± 23	98 ± 20	351 ± 46
	50	6.0	870±135	47±2	882±117	53 ± 4	892 ± 45	63 ± 5	1119 ± 66
**Absolute error**				11.03 ± 7.77 (mM)	23.94 ± 29.54 (Hz)	2.42 ± 2.53 (mM)	35.8 ± 29.3 (Hz)	65.19 ± 34.48 (mM)	58.2 ± 56.76 (Hz)
**Correlation with known concentrations or QUESP**				r=0.918 p<0.0001 CI=[0.790, 0.969]	r=0.980 p<0.0001 CI=[0.947, 0.993]	r=0.992 p<0.0001 CI=[0.977, 0.997]	r=0.971 p<0.0001 CI=[0.923, 0.989]	r=-0.161 p=0.522 CI=[-0.58, 0.33]	r=0.959 p<0.0001 CI=[0.891, 0.985]

CI = confidence interval

Supporting Information Table S3. AutoCEST determined semi-solid proton chemical exchange rates (k_{ssw}) and volume fractions (f_{ss}) for GM and WM brain tissue regions from three in vivo mice.

	**Semi-solid f_{ss} (%)**		**Semi-solid k_{SSW} (Hz)**	
Mouse #	Cortical GM	Sub-cortical WM[a]	Cortical GM	Sub-cortical WM[a]
1	12.21±1.37	19.73±3.30	60.81±9.28	46.23±14.70
2	12.27±2.50	19.23±3.84	55.25±12.72	40.64±19.76
3	13.82±1.81	20.44±3.32	53.55±11.20	44.75±15.17

Abbreviations: GM=gray matter, WM=white matter.
[a] Average of corpus callosum and white matter fiber tracts (cerebral peduncle, optic tract, and fimbria)[45,46].

Supporting Information Table S4. AutoCEST determined amide proton chemical exchange rates (k_{sw}) and volume fractions (f_s) for GM and WM brain tissue regions from an in vivo mouse.

	Cortical GM	Sub-cortical WM[a]
Amide k_{SW} (Hz)	61.03±29.24	73.02±51.11
Amide f_S (%)	0.29±0.16	0.40±0.27

Abbreviations: GM=gray matter, WM=white matter.
[a] Average of corpus callosum and white matter fiber tracts (cerebral peduncle, optic tract, and fimbria)[45,46].

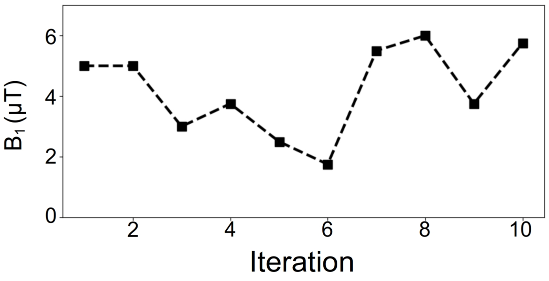

Supporting Information Figure S1. A previously reported phantom acquisition schedule[22], shortened to N=10 images and used as a reference unoptimized CEST-MRF protocol. The saturation pulse duration was 3s, the recovery time was 1s, the readout flip angle was 60°, and the saturation pulse frequency was set to the chemical shift of the exchangeable proton of each imaged phantom.

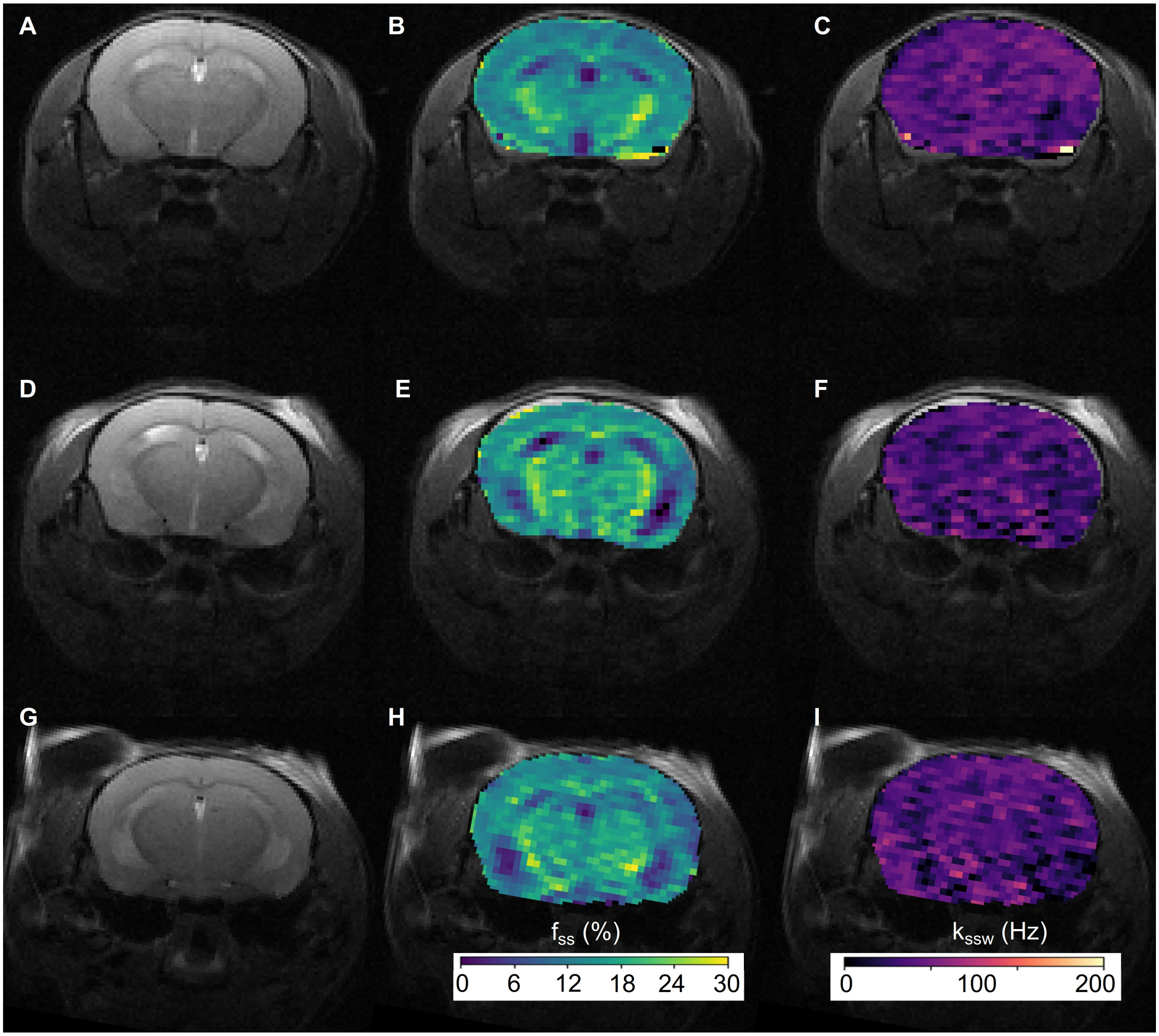

Supporting Information Figure S2. AutoCEST brain imaging of three in-vivo mice. Each row represents a different animal with T_2 -weighted images (A, D, G) and AutoCEST-generated semi-solid proton volume fraction (B, E, H) and chemical exchange rate maps (C, F, I).

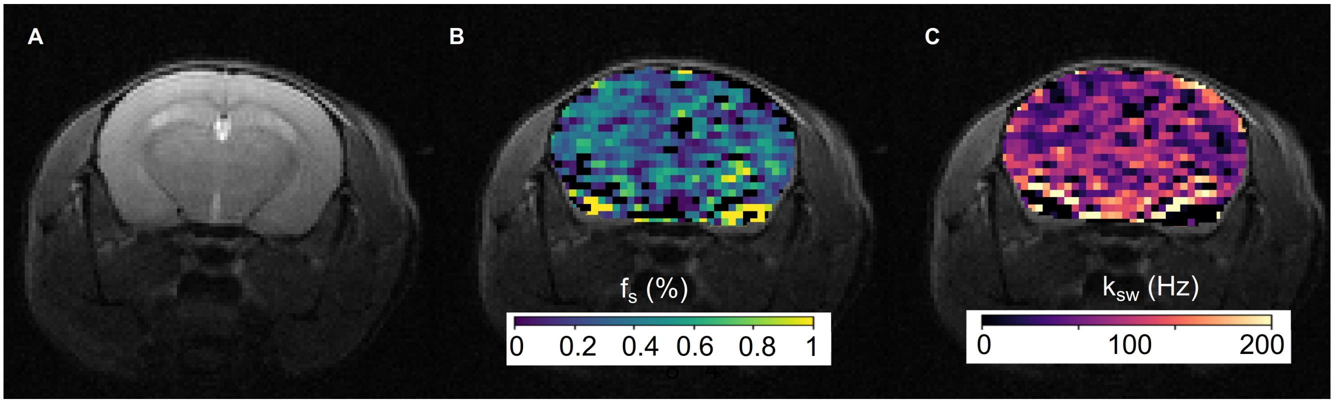

Supporting Information Figure S3. AutoCEST amide proton exchange parameter mapping of an in-vivo mouse. A. T_2-weighted image. B. AutoCEST-generated amide proton volume fraction (f_s). C. AutoCEST-generated amide proton chemical exchange rate (k_{sw}).

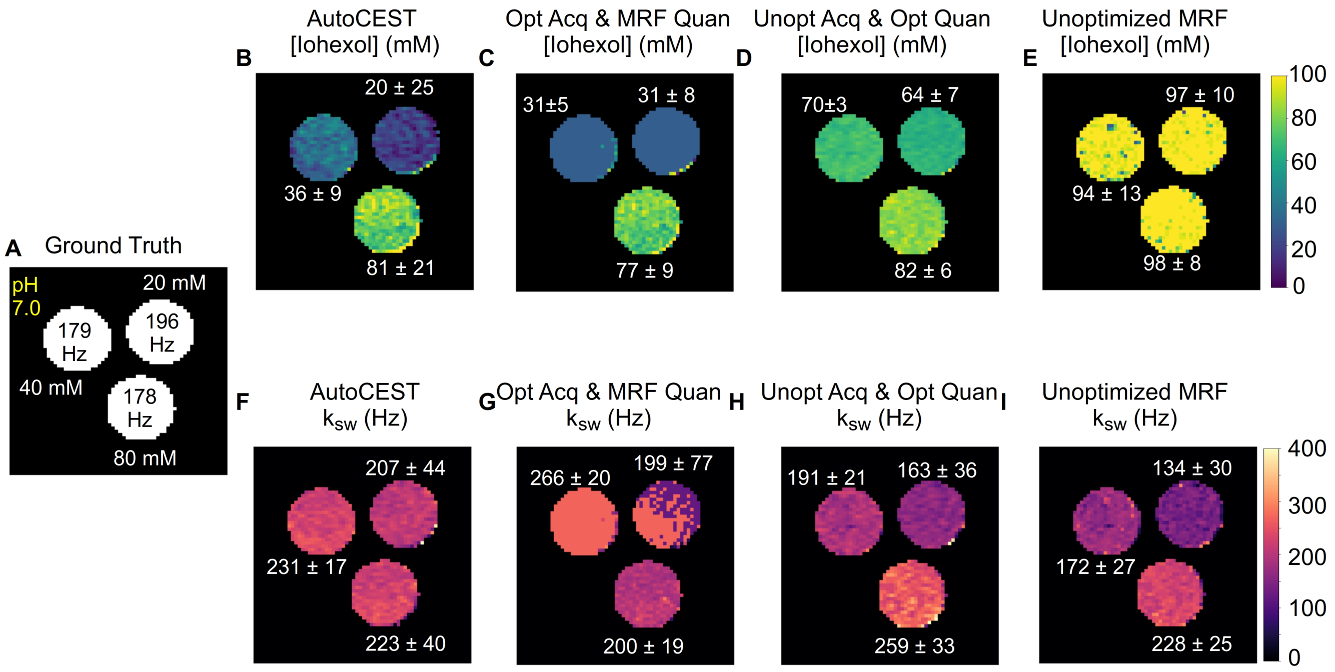

Supporting Information Figure S4. Comparison of different performance optimization methods – Iohexol phantom with various concentrations. (A). Ground truth concentrations and QUESP-determined proton exchange rates. The top row shows the resulting Iohexol concentration maps and the bottom row shows the resulting amide (4.3 ppm) proton exchange rate maps obtained using (B, F) autoCEST, (C, G) dot-product MRF quantification applied to data acquired using AutoCEST optimized schedules, (D, H) deep learning-based quantification applied to data acquired using an unoptimized CEST-MRF acquisition protocol, and (E, I) CEST-MRF dot-product quantification applied to an unoptimized acquisition schedule. The white text next to each vial represent its mean ± SD parameter value.

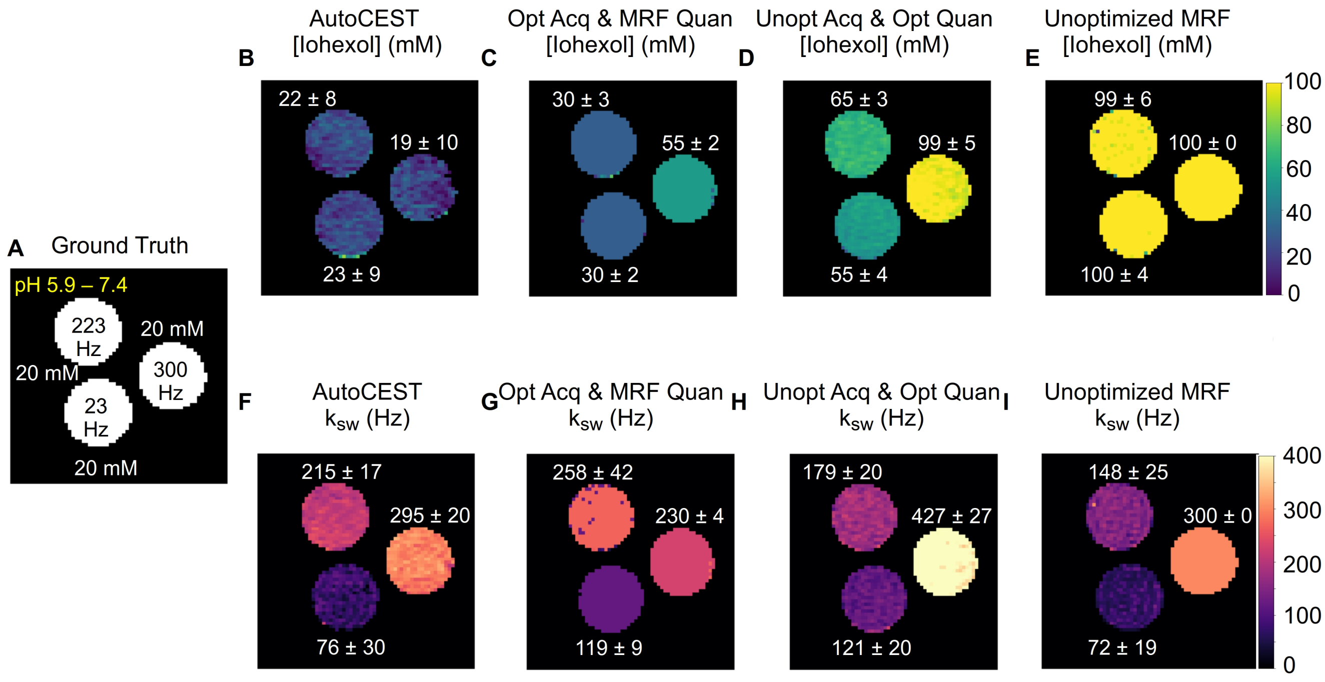

Supporting Information Figure S5. Comparison of different performance optimization methods – Iohexol phantom with various pH levels. (A). Ground truth concentrations and QUESP-determined proton exchange rates. The top row shows the resulting Iohexol concentration maps and the bottom row shows the resulting amide (4.3 ppm) proton exchange rate maps obtained using (B, F) autoCEST, (C, G) dot-product MRF quantification applied to data acquired using AutoCEST optimized schedules, (D, H) deep learning-based quantification applied to data acquired using an unoptimized CEST-MRF acquisition protocol, and (E, I) CEST-MRF dot-product quantification applied to an unoptimized acquisition schedule. The white text next to each vial represent its mean ± SD parameter value.

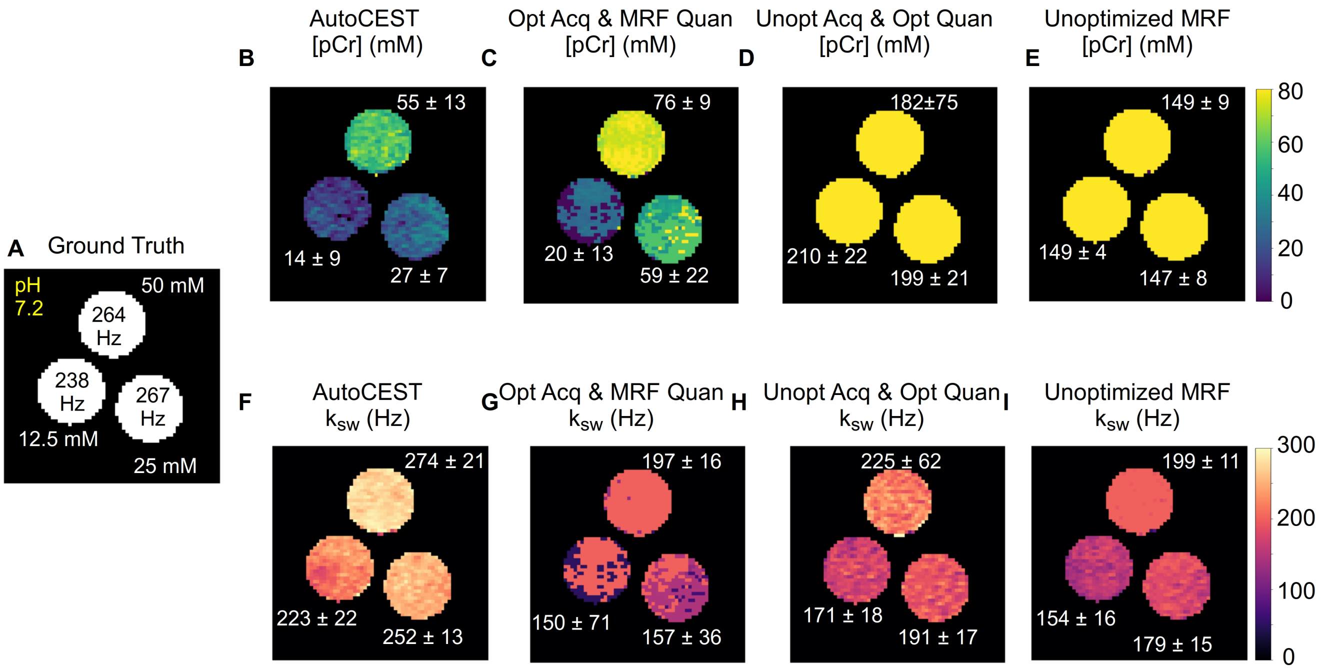

Supporting Information Figure S6. Comparison of different performance optimization methods – Phosphocreatine (pCr) phantom . (A). Ground truth concentrations and QUESP-determined proton exchange rates. The top row shows the resulting pCr concentration maps and the bottom row shows the resulting guanidinium (2.6 ppm) proton exchange rate maps obtained using (B, F) autoCEST, (C, G) dot-product MRF quantification applied to data acquired using AutoCEST optimized schedules, (D, H) deep learning-based quantification applied to data acquired using an unoptimized CEST-MRF acquisition protocol, and (E, I) CEST-MRF dot-product quantification applied to an unoptimized acquisition schedule. The white text next to each vial represent its mean ± SD parameter value.

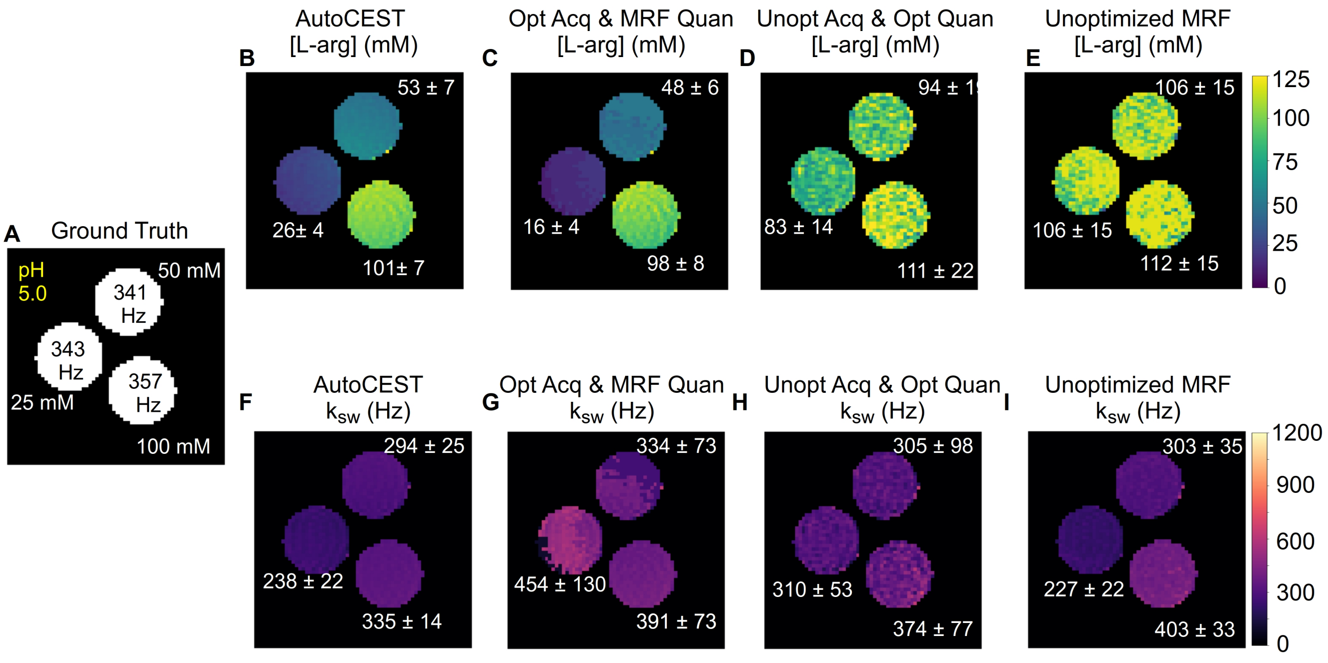

Supporting Information Figure S7. Comparison of different performance optimization methods – L-arginine phantom with various concentrations. (A). Ground truth concentrations and QUESP-determined proton exchange rates. The top row shows the resulting L-arginine concentration maps and the bottom row shows the resulting amine (3 ppm) proton exchange rate maps obtained using (B, F) autoCEST, (C, G) dot-product MRF quantification applied to data acquired using AutoCEST optimized schedules, (D, H) deep learning-based quantification applied to data acquired using an unoptimized CEST-MRF acquisition protocol, and (E, I) CEST-MRF dot-product quantification applied to an unoptimized acquisition schedule. The white text next to each vial represent its mean ± SD parameter value.

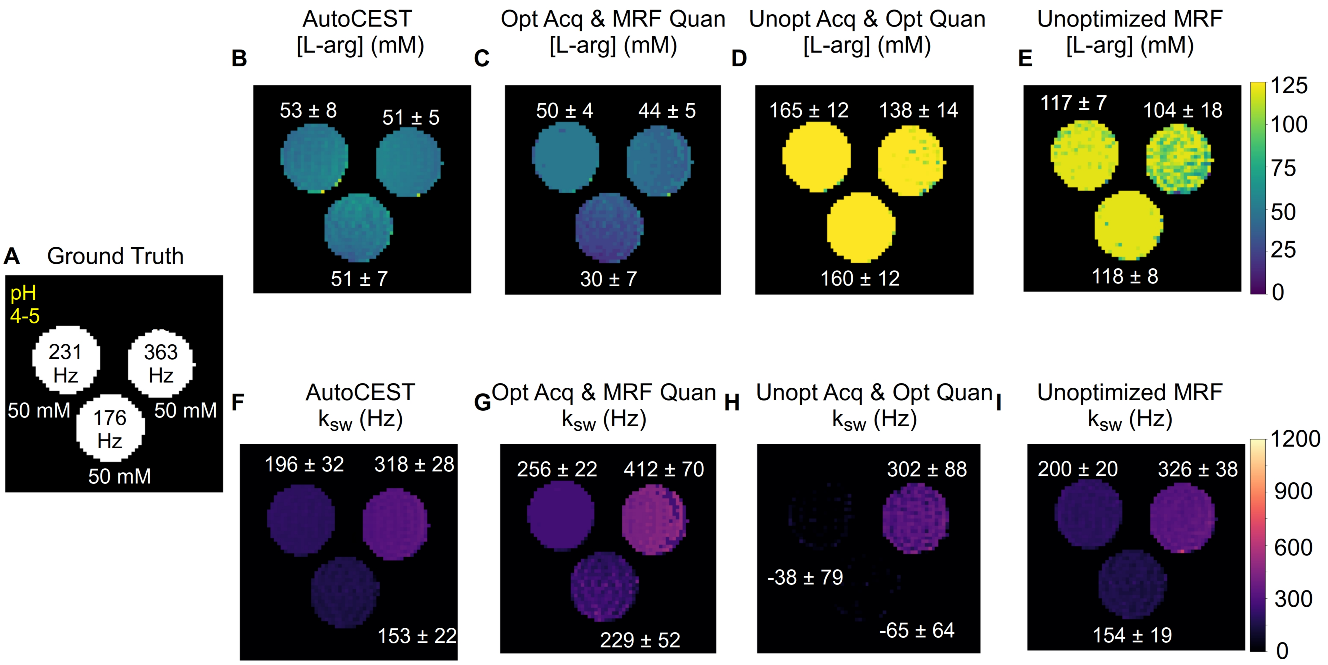

Supporting Information Figure S8. Comparison of different performance optimization methods – L-arginine phantom with pH 4-5. (A). Ground truth concentrations and QUESP-determined proton exchange rates. The top row shows the resulting L-arginine concentration maps and the bottom row shows the resulting amine (3 ppm) proton exchange rate maps obtained using (B, F) autoCEST, (C, G) dot-product MRF quantification applied to data acquired using AutoCEST optimized schedules, (D, H) deep learning-based quantification applied to data acquired using an unoptimized CEST-MRF acquisition protocol, and (E, I) CEST-MRF dot-product quantification applied to an unoptimized acquisition schedule. The white text next to each vial represent its mean ± SD parameter value.

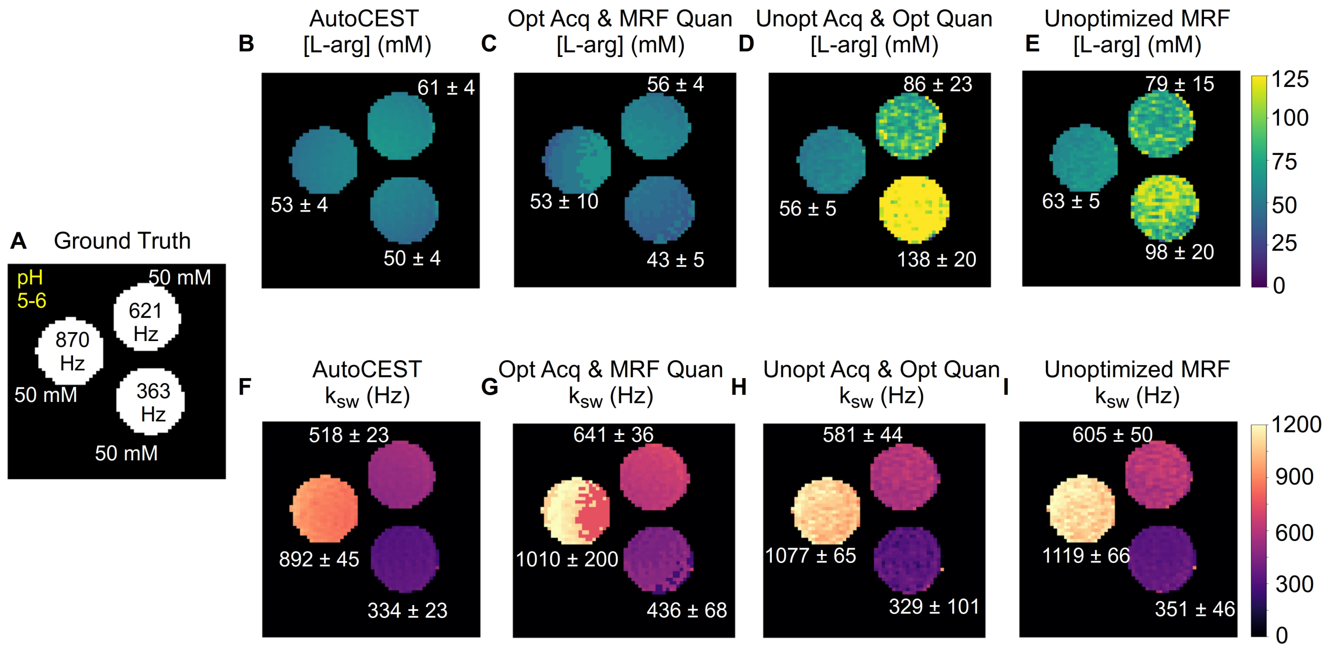

Supporting Information Figure S9. Comparison of different performance optimization methods – L-arginine phantom with pH 5-6. (A). Ground truth concentrations and QUESP-determined proton exchange rates. The top row shows the resulting L-arginine concentration maps and the bottom row shows the resulting amine (3 ppm) proton exchange rate maps obtained using (B, F) autoCEST, (C, G) dot-product MRF quantification applied to data acquired using AutoCEST optimized schedules, (D, H) deep learning-based quantification applied to data acquired using an unoptimized CEST-MRF acquisition protocol, and (E, I) CEST-MRF dot-product quantification applied to an unoptimized acquisition schedule. The white text next to each vial represent its mean ± SD parameter value.

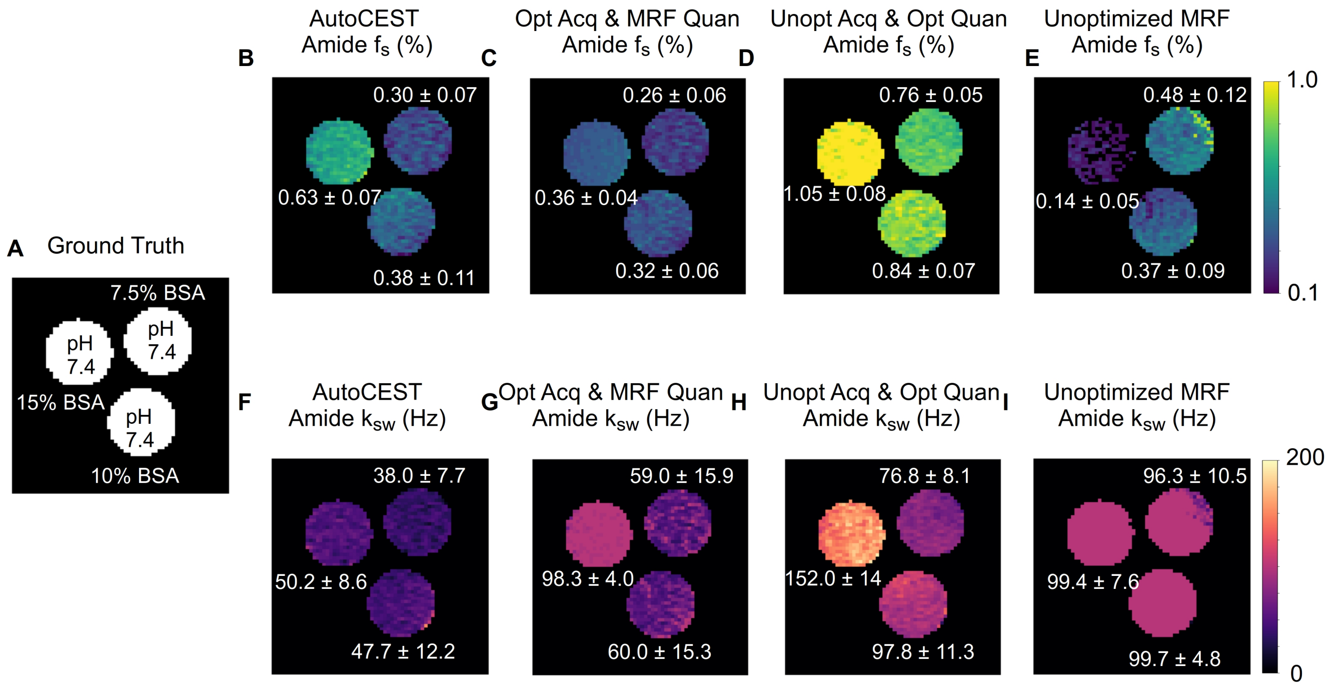

Supporting Information Figure S10. Comparison of different performance optimization methods – BSA phantom with amide (3.5 ppm) as the target compound. (A). Ground truth BSA concentrations and pH. The top and bottom rows show the resulting amide proton volume fraction and exchange rate maps, respectively, obtained using (B, F) autoCEST, (C, G) dot-product MRF quantification applied to data acquired using AutoCEST optimized schedules, (D, H) deep learning-based quantification applied to data acquired using an unoptimized CEST-MRF acquisition protocol, and (E, I) CEST-MRF dot-product quantification applied to an unoptimized acquisition schedule. The white text next to each vial represent its mean ± SD parameter value.

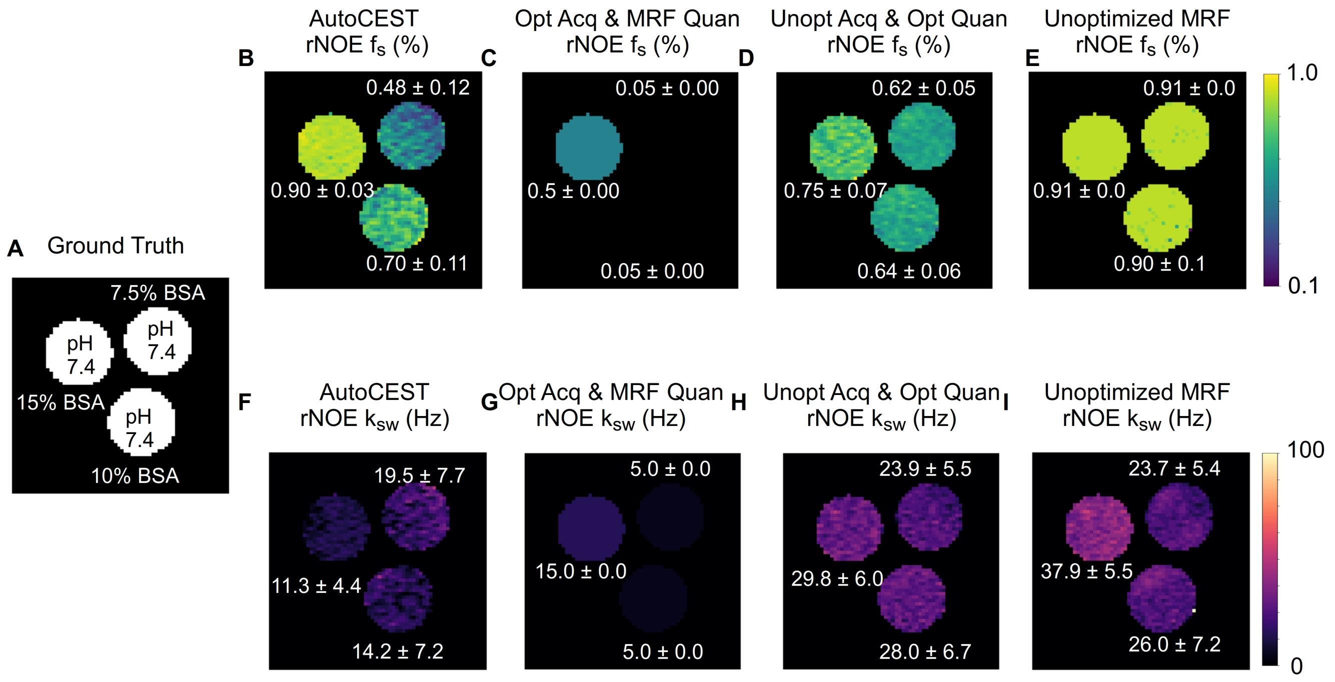

Supporting Information Figure S11. Comparison of different performance optimization methods – BSA phantom with aliphatic rNOE (-3.5 ppm) as the target compound. (A). Ground truth BSA concentrations and pH. The top and bottom rows show the resulting rNOE proton volume fraction and exchange rate maps, respectively, obtained using (B, F) autoCEST, (C, G) dot-product MRF quantification applied to data acquired using AutoCEST optimized schedules, (D, H) deep learning-based quantification applied to data acquired using an unoptimized CEST-MRF acquisition protocol, and (E, I) CEST-MRF dot-product quantification applied to an unoptimized acquisition schedule. The white text next to each vial represent its mean ± SD parameter value.

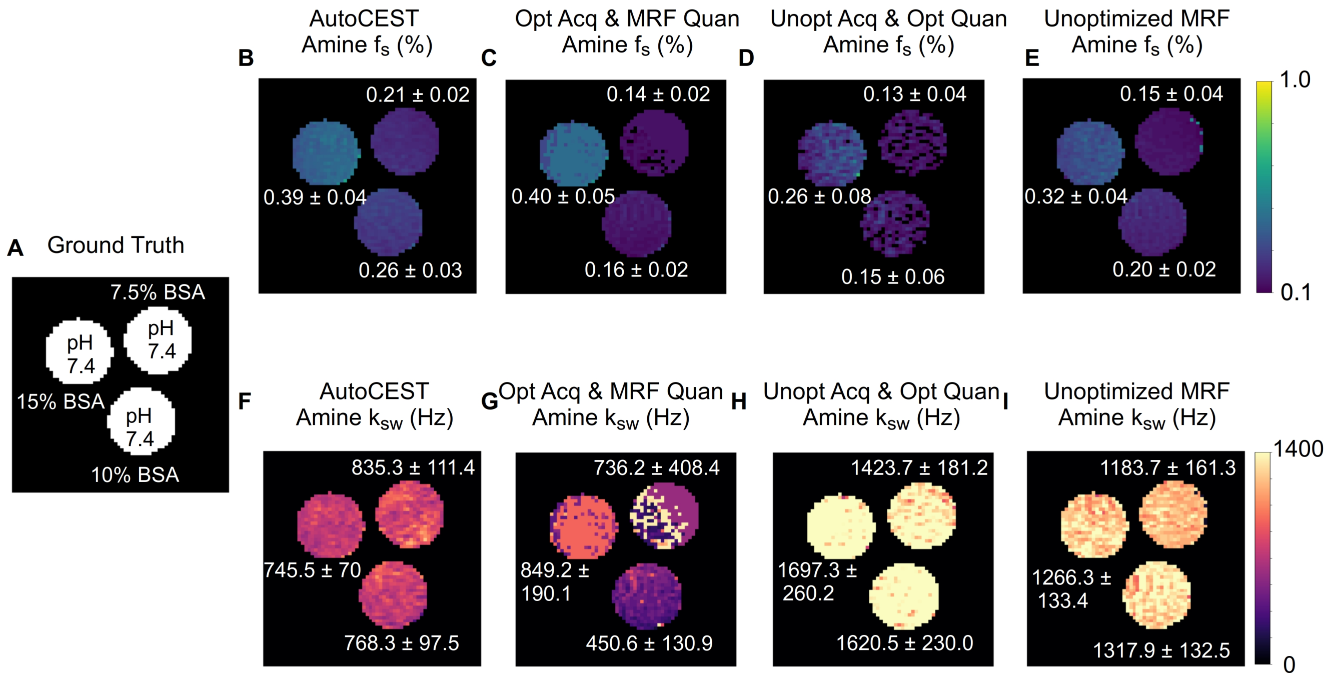

Supporting Information Figure S12. Comparison of different performance optimization methods – BSA phantom with amine proton (2 ppm) as the target compound. (A). Ground truth BSA concentrations and pH. The top and bottom rows show the resulting amine proton volume fraction and exchange rate maps, respectively, obtained using (B, F) autoCEST, (C, G) dot-product MRF quantification applied to data acquired using AutoCEST optimized schedules, (D, H) deep learning-based quantification applied to data acquired using an unoptimized CEST-MRF acquisition protocol, and (E, I) CEST-MRF dot-product quantification applied to an unoptimized acquisition schedule. The white text next to each vial represent its mean ± SD parameter value.

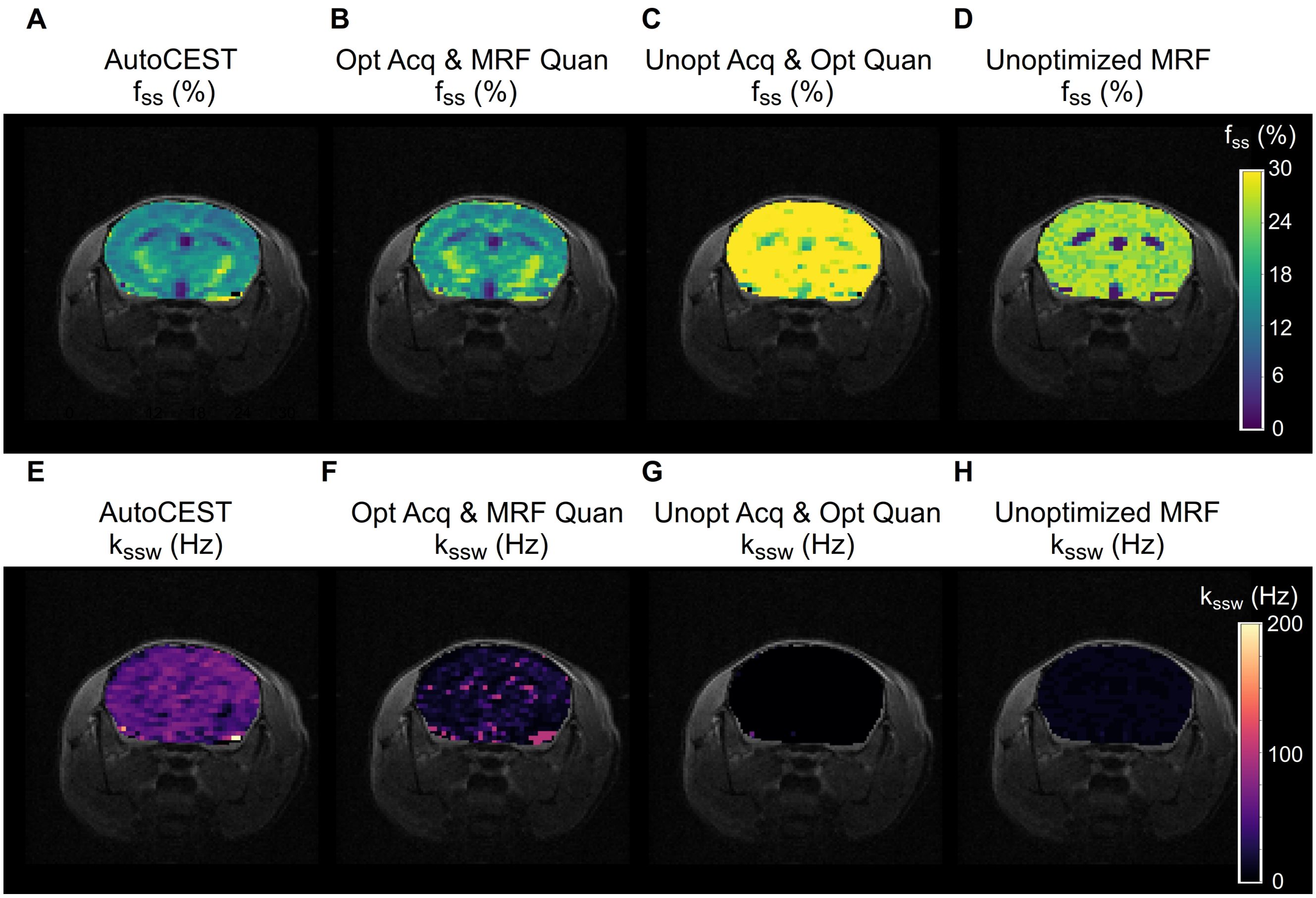

Supporting Information Figure S13. Comparison of different performance optimization methods – in vivo MT imaging, animal #1. The top and bottom rows show the resulting semi-solid proton volume fraction and chemical exchange rate maps, respectively, obtained using (A, E) autoCEST, (B, F) dot-product MRF quantification applied to data acquired using AutoCEST optimized schedules, (C, G) deep learning-based quantification applied to data acquired using an unoptimized CEST-MRF acquisition protocol, and (D, H) CEST-MRF dot-product quantification applied to an unoptimized acquisition schedule.

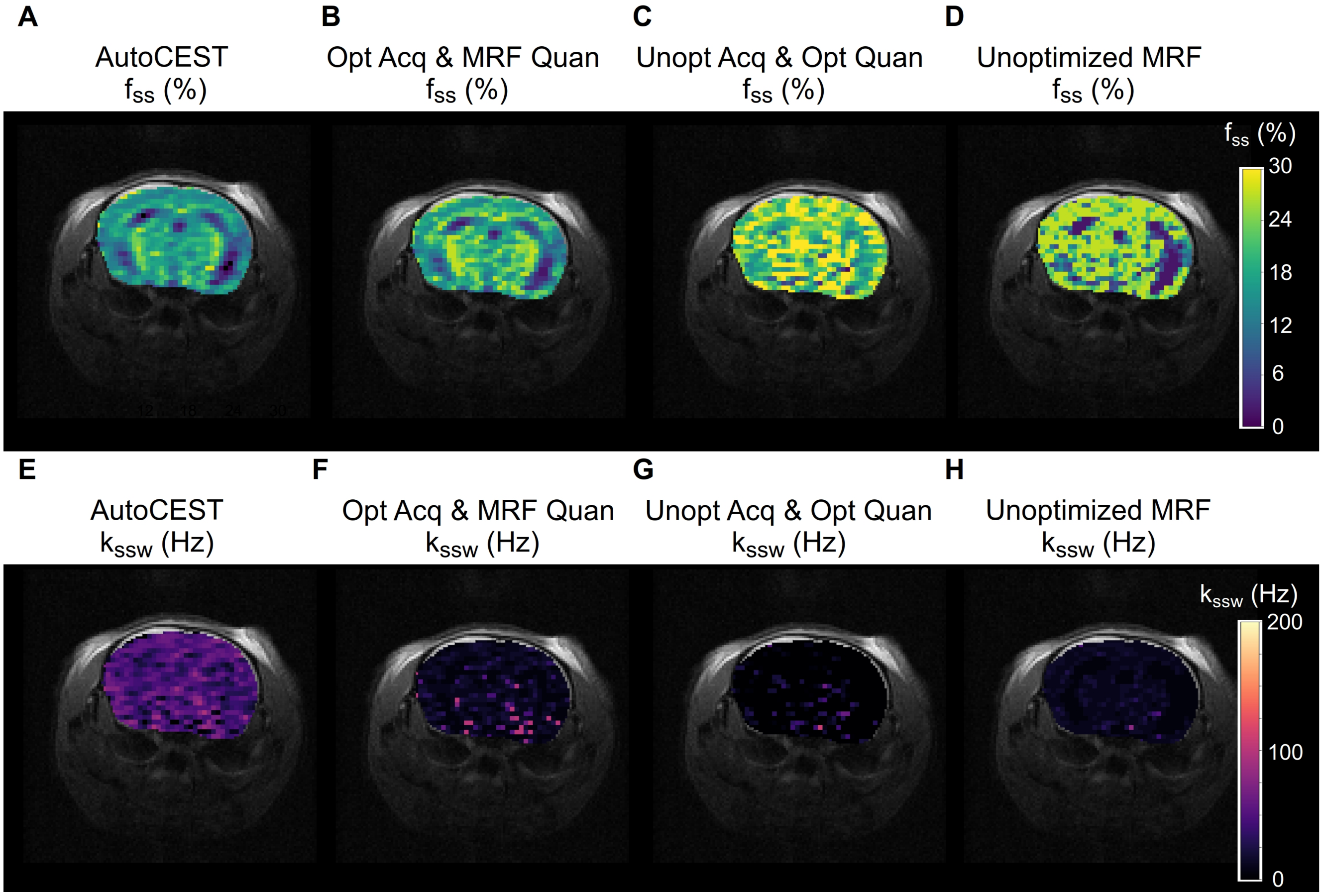

Supporting Information Figure S14. Comparison of different performance optimization methods – in vivo MT imaging, animal #2. The top and bottom rows show the resulting semi-solid proton volume fraction and chemical exchange rate maps, respectively, obtained using (A, E) autoCEST, (B, F) dot-product MRF quantification applied to data acquired using AutoCEST optimized schedules, (C, G) deep learning-based quantification applied to data acquired using an unoptimized CEST-MRF acquisition protocol, and (D, H) CEST-MRF dot-product quantification applied to an unoptimized acquisition schedule.

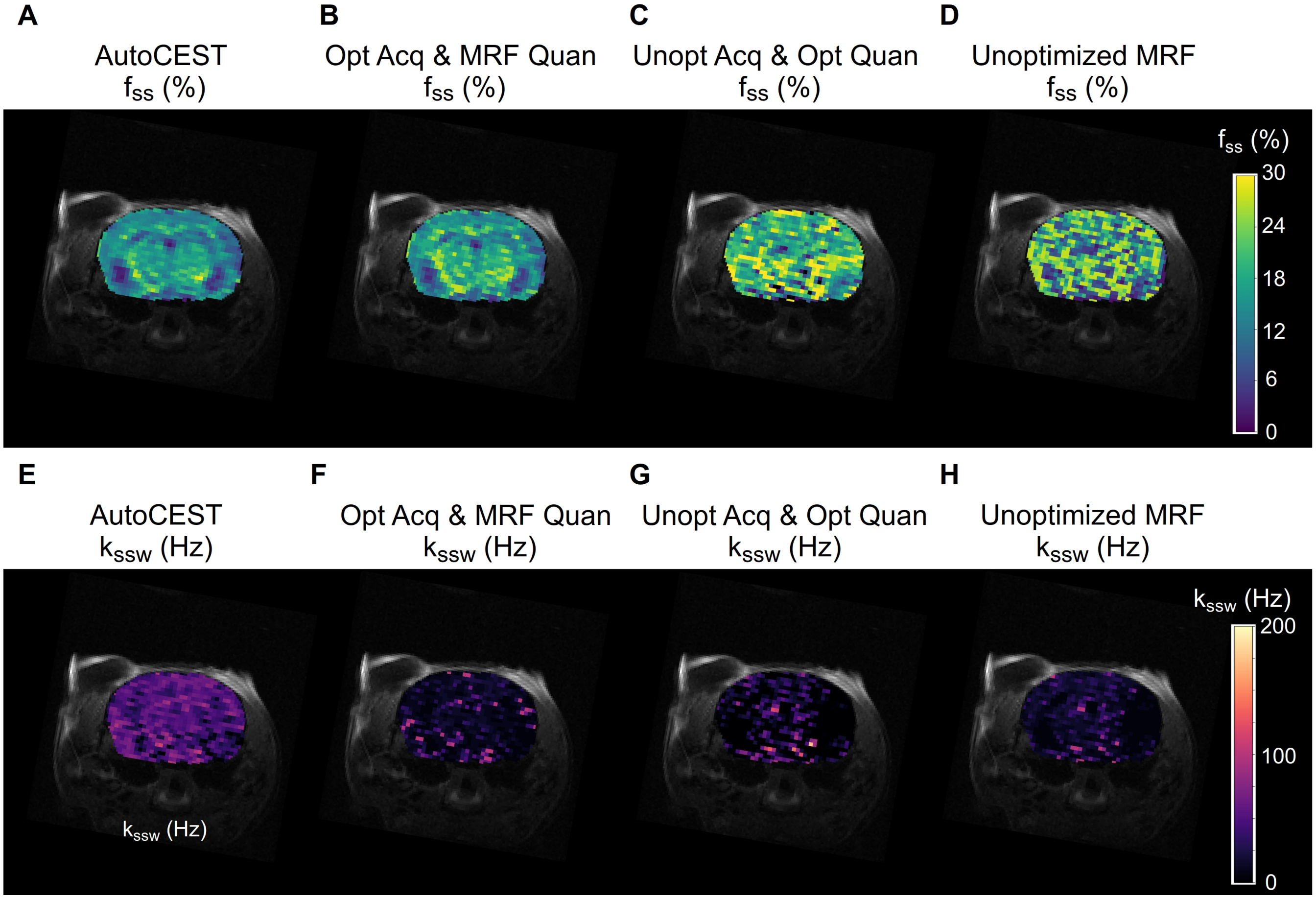

Supporting Information Figure S15. Comparison of different performance optimization methods – in vivo MT imaging, animal #3. The top and bottom rows show the resulting semi-solid proton volume fraction and chemical exchange rate maps, respectively, obtained using (A, E) autoCEST, (B, F) dot-product MRF quantification applied to data acquired using AutoCEST optimized schedules, (C, G) deep learning-based quantification applied to data acquired using an unoptimized CEST-MRF acquisition protocol, and (D, H) CEST-MRF dot-product quantification applied to an unoptimized acquisition schedule.

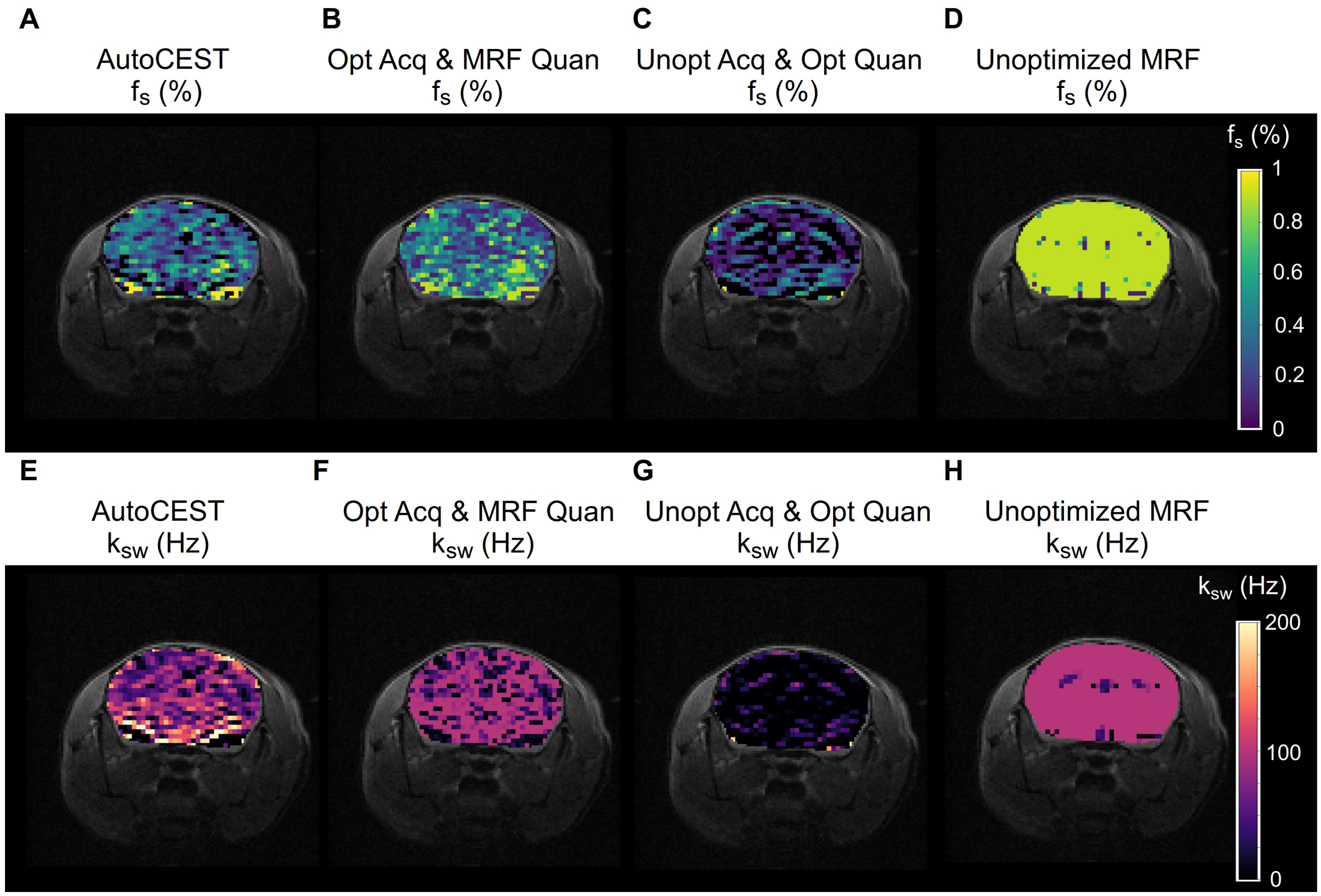

Supporting Information Figure S16. Comparison of different performance optimization methods – in vivo amide imaging. The top and bottom rows show the resulting amide proton volume fraction and chemical exchange rate maps, respectively, obtained using (A, E) autoCEST, (B, F) dot-product MRF quantification applied to data acquired using AutoCEST optimized schedules, (C, G) deep learning-based quantification applied to data acquired using an unoptimized CEST-MRF acquisition protocol, and (D, H) CEST-MRF dot-product quantification applied to an unoptimized acquisition schedule.
